# Three-dimensional reconstruction of lung tumors from computed tomography scans using adversarial and transductive learning

**DOI:** 10.1038/s41598-025-18899-7

**Published:** 2025-09-29

**Authors:** Zhisen He, Leila Jamel, Danyi Huang, Gaozhe Jiang, Zaffar Ahmed Shaikh, Md.Abeer Aljohani Khan, Seyed Jalaleddin Mousavirad

**Affiliations:** 1 Department of Electrical, Computer, and Systems Engineering, Case Western Reserve University, Cleveland, 44106 USA; 2 Department of Information Systems, College of Computer and Information Sciences, Princess Nourah bint Abdulrahman University, Riyadh, 11671 Saudi Arabia; 3 Department of Chemical Engineering, Columbia University, New York City, 10027 USA; 4 Department of Chemistry, The University of Hong Kong, Hong Kong, 999077 People’s Republic of China; 5 Department of Computer Science and Information Technology, Benazir Bhutto Shaheed University Lyari, Karachi, 75660 Pakistan; 6 School of Engineering, École Polytechnique Fédérale de Lausanne, Lausanne, 1015 Switzerland; 7 School of Biomedical Engineering, Hainan University, Haikou, 570228 China; 8 Department of Computer and Electrical Engineering, Mid Sweden University, Sundsvall, Sweden

**Keywords:** Lung cancer, Three-dimensional tumor reconstruction, Generative adversarial network, Off-policy proximal policy optimization, Transductive learning, Biomedical engineering, Computational science

## Abstract

Lung cancer is a critical health issue, and early detection is crucial for enhancing patient outcomes. This study presents a novel framework for generating three-dimensional (3D) representations of lung tumors from computed tomography (CT) scans, addressing three key challenges in the analysis process. Firstly, we address the precise segmentation of lung tissues, which is complicated by a high proportion of non-lung pixels that skew the classifier. Our method uses a customized generative adversarial network (GAN) enhanced with an off-policy proximal policy optimization (PPO) strategy. This strategy enhances segmentation performance by addressing inherent classifier biases and implementing a reward system to more accurately identify minority samples. Secondly, the framework enhances tumor detection in the segmented areas by employing a specialized GAN trained with an adversarial loss, which helps the generator create tumor regions that match real ones in both shape and internal features, even when contrast is low or boundaries are unclear. Thirdly, after tumor detection, the EfficientNet model extracts essential features for 3D reconstruction. The features are then enhanced by a spatial attention-based transductive long short-term memory (TLSTM) network for better performance. The TLSTM network enhances performance by assigning greater weight to samples near the test point within a transductive learning framework. Tested on the Lung Image Database Consortium Image Collection (LIDC-IDRI) dataset, our methodology achieved Hausdorff distance (HD) and Euclidean distance (ED) metrics of 0.648 and 0.985, respectively, indicating superior performance compared to existing methods. Our research introduces a clinical tool that significantly boosts the capabilities of radiologists in diagnosing and planning treatment for lung cancer. Code is publicly available at https://github.com/ZhisenHe/3D-representation/.

## Introduction

Lung cancer poses a significant threat to public health, being one of the most lethal cancers that results in a substantial number of fatalities globally each year. The World Health Organization (WHO) reports approximately 2.09 million new cases annually, culminating in around 1.76 million fatalities^1^. However, the early identification of lung cancer increases survival probabilities by up to 55%. Despite advanced imaging approaches such as CT scans, X-radiation (X-rays), and magnetic resonance imaging (MRI), intrinsic constraints still affect their utility^2,3^. CT scans produce precise 3D visuals of lung tissues, but their propensity for high false-positive rates often results in superfluous biopsies and operations^4–6^. X-rays are common for initial screenings, yet they lack the sensitivity to pinpoint tumors early and fail to accurately distinguish between benign and malignant growths. MRIs provide excellent soft tissue contrast but are less effective for lung imaging due to the substantial air content and motion of the organ during respiration^7^. These limitations highlight the need for more advanced computational techniques, especially those based on deep learning. Such methods can improve detection accuracy and support precise 3D reconstruction of lung tumors. Accurate 3D modeling is crucial for comprehending tumor morphology and informing effective treatment planning.

To date, advanced deep learning techniques have been applied to 3D modeling. Deep learning models are trained on large datasets of carefully labeled images. These models are highly effective at identifying complex patterns that often go unnoticed by human radiologists and traditional machine-learning techniques^8,9^. They improve the accuracy and reliability of lung cancer diagnosis. They also reduce false positives and limit the need for invasive procedures^10^. Deep learning models divide the task of 3D modeling into three subcomponents: lung segmentation, tumor detection, and 3D tumor reconstruction^2^. However, existing deep learning frameworks still face notable limitations in each of these stages. First, lung segmentation models often struggle with severe class imbalance, where non-lung pixels significantly outnumber lung pixels. This imbalance leads to biased classifiers that favor the majority class, thereby reducing segmentation accuracy. Second, despite architectural advances, accurate tumor detection remains difficult, especially when tumors have unclear boundaries or low contrast. This can result in both over-detection and missed detection of important regions. Third, many current 3D reconstruction methods rely on generalized temporal models such as standard LSTMs. These models often fail to capture subtle differences in tumor growth or spatial structure, which leads to low-quality reconstructions and limited clinical usefulness^10^. To address the specific challenges in lung segmentation, tumor detection, and 3D reconstruction, this study proposes a unified deep-learning framework that integrates all three modules to generate high-fidelity 3D representations of lung tumors.

Traditional methods for addressing class imbalance in lung segmentation operate at two levels. At the data level, techniques include undersampling the majority class, oversampling the minority class, and generating synthetic samples. At the algorithmic level, loss functions are reweighted to emphasize signals from the minority class. Although these strategies are partially effective, they often lead to overfitting, poor generalizability, and limited adaptability to complex spatial patterns in medical images^11^. Deep reinforcement learning (DRL) has recently emerged as a promising alternative. It filters noise, sharpens critical features, and has proven effective in various domains. In DRL, the reward function can mitigate class imbalance by assigning higher rewards for the correct classification of minority-class samples and applying stronger penalties for their misclassification. This weighting scheme encourages the learning algorithm to prioritize underrepresented classes, thereby reducing bias toward the majority class and enhancing overall fairness in recognition^12^. However, DRL methods often face the bias-variance trade-off, which can lead to unstable training and inconsistent results. In this paper, we adopt PPO to handle the imbalance problem in lung segmentation more effectively. PPO is an on-policy RL algorithm that stabilizes learning by preventing large policy updates through a clipping mechanism. It maintains a balance between exploration and exploitation and is computationally efficient for high-dimensional tasks with continuous variables. To improve performance and data efficiency, we use an off-policy version of PPO. This variant reuses agent-environment interactions stored in a replay buffer, which reduces the cost of training and enhances generalization in complex, high-dimensional environments such as medical image segmentation^13^.

To address the challenges of accurate tumor detection, particularly in regions with unclear boundaries or low contrast, our model incorporates a composite loss function consisting of classification loss, bounding box regression loss, and a novel adversarial loss component. Most conventional methods use only classification and bounding box regression losses for tumor classification and localization^10^. In contrast, our method includes adversarial learning to enhance feature-level realism and structural precision. The adversarial loss serves as a powerful regularizer, encouraging the generator to produce bounding boxes that are both geometrically accurate and feature-wise similar to those of real tumor regions. The discriminator compares the feature maps of predicted and actual tumor regions to provide detailed feedback. This feedback improves the ability of the generator to identify tumor-like regions, even when texture contrast is low or boundaries are unclear. This integration of adversarial loss effectively overcomes the limitations of conventional detection methods, which often fail under subtle or ambiguous visual conditions. As a result, our model delivers more reliable tumor localization and greatly enhances the clinical utility of the final 3D reconstructions.

After addressing challenges in lung segmentation and tumor detection, the next challenge lies in improving temporal modeling during 3D tumor reconstruction. We utilize a TLSTM network^14^ to address the limitations of existing temporal models in 3D tumor reconstruction. TLSTM adjusts its parameters based on the proximity and unique features of each input sample. Unlike conventional LSTM models that apply the same learning to all data, TLSTM dynamically adapts to the test data distribution. For example, standard LSTM models may not distinguish between slow-growing and fast-growing tumors when progression rates vary. In contrast, TLSTM adapts more effectively to these local differences. We also include a spatial attention mechanism in TLSTM. This enables the model to focus on the most important parts of the input sequence, improving the accuracy and clinical relevance of the 3D reconstructions.

After addressing the three core challenges separately, we now present the overall architecture of the proposed framework. This framework integrates the segmentation, detection, and reconstruction modules into a unified 3D modeling pipeline. The article introduces a triple-tiered strategy for creating 3D representations of lung cancer from CT scans. Initially, we employ a GAN featuring a U-shaped network (U-Net) architecture to segment the CT images. To address the class imbalance problem, we use the off-policy PPO approach. The discriminator applies dilated convolution layers to improve the generator output. It distinguishes real images from artificial ones and emphasizes essential features during training. In the second phase, the segmented images are processed using another GAN configured with the mask region-based convolutional neural network (Mask R-CNN) architecture. This network accurately identifies and locates target areas within the images for precise tumor detection. To reinforce the accuracy of tumor localization, the generator applies a specific loss function, ensuring alignment between synthetic outputs and actual CT data. The discriminator supports output validation using dilated convolutions for robust feedback. The final phase involves 3D modeling, where a spatial attention-based TLSTM network is paired with a GAN. At this stage, the tumor images from the second step are fed into the trained EfficientNet network to extract features. These features are then passed to the TLSTM and subsequently to the generator, which constructs a 3D model based on the TLSTM outputs.

The effectiveness of our approach is robustly confirmed via extensive evaluation, and scrupulously compared with established methodologies utilizing the LIDC-IDRI dataset and standard performance indicators. This detailed juxtaposition underscores the superior performance and promising potential of our method to enhance the early detection of lung cancer. Table 1 lists the abbreviations and their corresponding definitions used throughout the study.

The critical contributions of our model include:

- Our model employs off-policy PPO to tackle the class imbalance commonly found in traditional lung segmentation methods. Off-policy PPO differs from on-policy methods as it utilizes data from various policies, which boosts the efficiency of the model. This feature is especially useful in medical contexts where data from infrequent but crucial conditions are limited. Additionally, we have incorporated a novel reward system within the off-policy PPO framework. This system focuses on the minority class by rewarding accurate identification and penalizing errors in classifying underrepresented classes, thereby encouraging a more balanced and fair classification process.
- A novel adversarial loss term is integrated into the generator loss function during tumor detection. Conventional methods rely only on classification and bounding box regression losses. In contrast, the adversarial component enhances the feature-level realism of predicted regions. This helps the model detect tumors more effectively, even when contrast is low or boundaries are unclear. This enhancement significantly reduces false detections and improves clinical reliability.
- The TLSTM method represents a substantial innovation in 3D tumor modeling. It enhances model training by weighing the influence of training samples based on their proximity to test samples. This approach significantly improves the precision and consistency in 3D representations, making it a valuable tool for accurate tumor morphology analysis.

The structure of this article is as follows: relevant research on lung cancer is reviewed next, followed by the basic concepts. The proposed methodology is then outlined, after which the findings and analysis of our experiments are presented. Finally, the paper concludes with closing remarks. 

**Table 1:** List of abbreviations and their definitions used in the study.

Abbreviation	Definition
3D	Three-dimensional
CT	Computed tomography
GAN	Generative adversarial network
PPO	Proximal policy optimization
TLSTM	Transductive long short-term memory
LIDC-IDRI	Lung image database consortium image collection
HD	Hausdorff distance
ED	Euclidean distance
WHO	World Health Organization
X-rays	X-radiation
MRI	Magnetic resonance imaging
DRL	Deep reinforcement learning
U-Net	U-shaped network
Mask R-CNN	Mask region-based convolutional neural network
AI	Artificial intelligence
U-Net++	U-shaped++
TB	Tuberculosis
LDANet	Lung-dense attention network
RSA	Residual spatial attention
GCA	Gated channel attention
DAGM	Dual attention guidance module
LDB	Lightweight dense block
PTB	Positioned transpose block
CXR	Chest X-ray
Xception	Extreme inception
ResNet-18	Residual network-18
COVID-19	Coronavirus disease 2019
FL	Federated learning
SSSOA	Salp shuffled shepherd optimization algorithm
VGG16	Visual geometry group 16
CAD	Computer-aided diagnostic
EfficientNet B3	Efficient network B3
T-Net	T-shaped network
CenterNet	Center-based object detection network
NASNet	Neural architecture search network
TFDM	Differential memory
WOA	Whale optimization algorithm
SVM	Support vector machine
CapsNet	Capsule neural network
WSTSA	Wormhole and Salp swarm strategy enhanced tree-seed algorithm
HM-LeNet	Hybrid mobile LeNet
SNP	Single nucleotide polymorphism
LDN	Lightweight deep network
ANN	Artificial neural network
ROI	Region of interest
2D	Two-dimensional
GGO	Ground glass opacity
CBAM	Convolutional block attention module
ASPP	Atrous spatial pyramid pooling
ReLU	Rectified linear unit
DSC	Dice similarity coefficient
FSIM	Feature similarity index measure
APSO	Adaptive particle swarm optimization
XGBoost	Extreme gradient boosting
HRCT	High-resolution computed tomography
IoT	Internet of things
OFCMNN	Optimized fuzzy C-means neural network
KM-DTCL	Kernel multilayer deep transfer convolutional learning
Coarse Seg-net	Coarse segmentation subnetwork
Fine Seg-net	Fine segmentation subnetwork
Class-net	Classification subnetwork
ROC	Receiver operating characteristic
AUC	Area under the curve
HRNet	High-resolution network
DLN	Deep learning nomogram
ITF	Intrathoracic fat
IPN	Intranodular and perinodular regions
LASSO	Least absolute shrinkage and selection operator
PET	positron emission tomography
KAN	Kolmogorov–Arnold networks
SE	Squeeze-and-excitation
ViT	Vision transformer
Grad-CAM	Gradient-weighted class activation mapping
YOLOv8	You Only Look Once version 8
DCGAN	Deep convolutional generative adversarial network
FPN	Feature pyramid network
MSDA	Multi-scale dilation attention
GCSAM	Global channel spatial attention mechanism
CNDNet	Candidate nodule detection network
FPRNet	False positive reduction network
HPFF	Hierarchical progressive feature fusion
LUNA16	Lung nodule analysis 2016
GK	Gustafson and Kessel
TRPO	Trust region policy optimization
RNN	Recurrent neural network
MDP	Markov decision process
KL	Kullback-Leibler
EMD	Earth Mover’s distance
BN	Batch normalization
FNIH	Foundation for the national institutes of health
FDA	Food and drug administration
XML	Extensible markup language
IoU	Intersection over union
RPN	Region proposal network
ADAM	Adaptive moment estimation
GPU	Graphics processing unit
GB	Gigabyte
FGSM	Fast gradient sign method

## Related works

The advent of artificial intelligence (AI) has dramatically transformed various medical fields^15–17^, enhancing diagnostic performance and treatment results in areas including skin, colon, and heart cancers^18–23^. Lung cancer, notable for its high prevalence and mortality, requires sophisticated technological solutions for timely detection and accurate treatment planning^24–27^. Specifically, creating 3D representations of lung tumors is essential to grasp tumor growth complexities and improve treatment methods.

This review meticulously divides the literature on lung cancer detection and analysis into three key sections: lung segmentation, tumor detection, and 3D tumor reconstruction. Each section discusses significant developments and methods that enhance diagnostic and treatment techniques in lung cancer care.

### Lung segmentation

In 2023, Gite et al.^28^ explored the effectiveness of U-shaped network++ (U-Net++) for lung segmentation in X-ray images to enhance tuberculosis (TB) detection. Their approach marks a significant departure from traditional methods by segmenting lungs prior to classification, which is shown to increase TB detection performance. The study compares U-Net++ with three other segmentation models, underscoring the advantages of using advanced segmentation before classification to prevent data leakage and improve diagnostic outcomes. Arvind et al.^29^ have enhanced the U-Net architecture to develop a lighter, more efficient version for semantic lung segmentation from chest X-ray images. This modified U-Net includes multiple dropouts in all deconvolutional layers, effectively addressing overfitting and surpassing the performance of the original model. The redesigned architecture demonstrates robust generalization across diverse datasets, proving particularly effective in quickly identifying thoracic abnormalities and thereby facilitating prompt and accurate medical diagnoses. Chen et al.^30^ proposed a novel lung-dense attention network (LDANet) to automatically segment lung parenchyma from CT images. This network features two main mechanisms: residual spatial attention (RSA) and gated channel attention (GCA). RSA emphasizes spatial details and suppresses irrelevant areas, while GCA adaptively adjusts channel weights to highlight critical features. Additionally, LDANet uses a dual attention guidance module (DAGM) to integrate features better and combines a lightweight dense block (LDB) with a positioned transpose block (PTB) to reuse features effectively and restore image resolution accurately. Ghali et al.^31^ developed a system for accurately segmenting lungs in chest X-ray (CXR) images, which is crucial for enhancing automated analysis and assisting radiologists. The proposed method utilizes five different models to delineate lung regions effectively, even in challenging cases involving varied lung shapes and disease-affected tissues. This approach incorporates dual loss functions to optimize the segmentation process, thereby improving the diagnostic reliability for healthy and unhealthy lungs. Murugappan et al.^32^ explored a robust deep-learning approach for lung segmentation in CT images using the DeepLabV3 + network. The study employs various pre-trained networks such as Extreme Inception (Xception), Residual Network-18 (ResNet-18), and ResNet-50, refining the segmentation for two-class and four-class distinctions. The approach significantly enhances the delineation of lung fields and infected regions, which is crucial for effectively diagnosing Coronavirus disease 2019 (COVID-19). Ambesange et al.^33^ developed an AI model using a federated learning (FL) framework combined with transfer learning to segment lung X-ray images efficiently. This approach maintains data privacy by training on local datasets and without requiring data centralization. The model employs pre-trained U-Net weights and utilizes local healthcare data to enhance training, further supported by explainable AI techniques to clarify model decisions.

In 2024, Hasan et al.^34^ utilized the Deeplabv3plus CNN-based model to segment lung regions from X-ray images for enhanced tuberculosis detection. This model addresses the common issue of reduced feature resolution in deep networks by integrating atrous convolution and modifying pooling and convolutional striding mechanisms. This adaptation allows for more precise lung segmentation and optimizes the model for specific diagnostic tasks in pulmonary disease detection. Swaminathan et al.^35^ addressed late-stage lung cancer diagnosis by implementing deep learning for earlier detection in high-risk patients. They enhanced CT images with a Wiener filter, segmented them using a GAN trained with the Salp Shuffled Shepherd Optimization Algorithm (SSSOA), and then classified the segments with the Visual Geometry Group 16 (VGG16) CNN model. This complex multi-step process may introduce computational inefficiencies or require fine-tuning for different datasets. Suji et al.^36^ developed a computer-aided diagnosis (CAD) system utilizing deep learning for lung cancer detection, specifically focusing on lung nodule segmentation. The method uses transfer learning to customize pre-trained weights from different encoders in an encoder-decoder framework and explores various combinations to find the best setup. The selected architecture, U-Net with an EfficientNet B3 backbone, was tailored for performance on the LIDC-IDRI lung cancer dataset. Thangavel and Palanichamy^37^ introduced an advanced deep-learning framework for classifying pulmonary nodules using CT images, addressing the challenges of diverse and visually similar lung nodules. The approach integrates a T-shaped network (T-Net) for segmentation, a center-based object detection network (CenterNet) for extracting texture and intensity attributes, and a Neural Architecture Search network (NASNet) for classification, thereby ensuring thorough analysis without manual feature integration. This method leverages multiple preprocessing techniques to enhance data quality for effective learning and classification. Cai et al.^38^ proposed a novel lung CT image segmentation method utilizing GANs. This method leverages the image translation capabilities of various GANs to transform original lung images into segmented outputs, aiming to enhance the effectiveness of pulmonary disease diagnosis by providing clear segmentation of lung tissues. Ramos and Pineda^39^ developed a tri-phase, semi-automated lung CT image segmentation pipeline. This method initiates with preprocessing techniques such as contrast enhancement and noise reduction, followed by region growing for segmentation, and concludes with morphological closing to refine results. The approach is designed to provide precise segmentation without relying on extensive training datasets, making it particularly valuable in settings with limited data resources. Zheng et al.^40^ introduced an advanced lung segmentation algorithm designed to tackle the complexities of lung images characterized by strong noise and uneven grayscale distribution. The method starts by extracting an initial lung mask using threshold techniques and gradient extraction with a time-series method called time-series differential memory (TFDM). Additionally, the approach incorporates an improved convex hull method for lung contour repair, addressing issues like contour loss due to solid nodules and other lesions. Guo et al.^41^ introduced an improved method for segmenting lung cancer pathological images. This method utilizes the whale optimization algorithm (WOA) and a random mutation strategy to enhance image segmentation. This approach is tailored to boost convergence speed and to circumvent local optima. The method is refined using a multilevel segmentation strategy tailored to lung cancer analysis. Pandey and Bhandari^42^ proposed a method for early detection of lung cancer using CT images, combining morphological filtering and contour-based image segmentation techniques to identify malignant tumors in their initial stages. Following segmentation, a Support Vector Machine (SVM) classifier differentiates malignant lung CT scan images from non-malignant ones.

In 2025, Vijayakumar et al.^43^ developed a machine learning-based model to detect lung cancer using an advanced classifier and innovative segmentation methods. The process starts with preprocessing CT images using rolling guidance filtering and a switched mode fuzzy median filter, followed by segmentation with U-net. The segmented images are analyzed using a Capsule Neural Network (CapsNet), which preserves spatial relationships effectively and reduces feature loss, leading to faster and more efficient training. Qiao et al.^44^ introduced a novel approach combining a wormhole and salp swarm strategy enhanced tree-seed algorithm (WSTSA) with a genetic algorithm for neural architecture search in medical image segmentation. This method automates the design of deep learning models, optimizes hyperparameters, and explores optimal network architectures to improve segmentation performance. The integration of WSTSA enhances the efficiency of architecture search, enabling the development of high-performing convolutional neural networks tailored for medical imaging tasks. Li et al.^45^ proposed an advanced lung CT image segmentation framework that combines an attention mechanism with sobel edge detection to enhance the quality of lesion delineation. This method uses residual dynamic convolutions to enhance feature extraction. It includes an information fusion attention module that refines features with attention mechanisms and dilated convolutions, improving edge clarity and segmentation sharpness. Murugaraj et al.^46^ developed a hybrid mobile LeNet (HM-LeNet) that integrates aspects of MobileNet and LeNet for enhanced segmentation and classification of lung vessels in CT images. This method begins with preprocessing using a non-local means filter, followed by lung lobe segmentation with K-Net and pulmonary vessel segmentation. The HM-LeNet then classifies lung abnormalities, such as emphysema, nodules, and pulmonary embolisms, leveraging advanced feature extraction capabilities. Fu et al.^47^ introduced the single nucleotide polymorphism (SNP)-based lightweight deep network (LDN-SNP) model for segmenting COVID-19 CT images. This model features a novel neuron design known as SNP-type neurons. This model features a compact architecture with only 81,316 parameters, designed to overcome the challenges of limited labeled samples and computational inefficiency common in larger models. The network structure consists of an encoder using three specialized convolution modules and a decoder with a fourth module, achieving efficient training and deployment capabilities for practical medical use. Erciyes et al.^48^ presented a deep learning method that utilizes an artificial neural network (ANN) in conjunction with preprocessing, adaptive augmentation, and an improved convolutional encoder-decoder based on a U-Net to address overfitting and enhance boundary delineation. Yang et al.^49^ developed a 3D lightweight segmentation model that integrates region of interest (ROI) extraction via K-means clustering, attention-enhanced skip connections, and a combination of focal loss and the dice coefficient. The model also employs depth-wise separable convolutions, ghost-inspired 1 × 1 convolutions, and residual connections. Nguyen et al.^50^ proposed a two-stage segmentation approach combining two-dimensional (2D) and 3D neural networks based on no-new-U-Net, enhanced with attention mechanisms and boundary loss to improve segmentation of ground glass opacity (GGO) in post-COVID-19 CT scans. Vincy et al.^51^ introduced a segmentation model using a 3D ResNet50 to encode lung CT slices. The model employs a dense feature extraction module and a U-Net decoder for multiscale extraction of tumor features. A combined Dice and focal loss were applied to address class imbalance. Jannat et al.^52^ suggested a deep learning model called the lightweight residual U-Net, which integrates a convolutional block attention module (CBAM), atrous spatial pyramid pooling (ASPP), and attention mechanisms, trained using rectified linear unit (ReLU) and leaky ReLU activations. Notably, they found that the dice loss outperformed other losses.

Table 2 provides a comprehensive review of the most sophisticated models for lung segmentation. Most existing approaches adopt U-Net variants^28,29,36,48^, attention mechanisms^30,45^, or GAN-based frameworks^38^ to classify each pixel as lung or non-lung. However, a shared limitation across these models is their restricted capacity to handle severe class imbalance, where non-lung pixels vastly outnumber lung pixels in CT or X-ray images. This often leads to biased classifiers that underperform on minority classes, particularly in atypical or diffuse lung regions. Some studies employ types of loss terms like dice loss and focal loss^31,49–52^ to more accurately represent minority classes. However, these methods are often sensitive to image quality and depend on manually tuned parameters.

Our proposed method distinguishes itself by embedding an off-policy PPO strategy into a GAN-based segmentation framework. Unlike traditional static weighting methods, the PPO-based agent uses a reward system to learn how to focus on underrepresented lung regions and improve minority-class accuracy. The off-policy design enables the model to reuse past training experiences, thereby improving stability and generalization —a capability that is rarely addressed in existing segmentation methods. This reinforcement-driven segmentation process not only reduces bias but also avoids overfitting and the need for manual threshold tuning.

**Table 2:** Comparison of advanced models for lung segmentation.

Authors	Methodology	Contribution	Dataset	Result	Limitation
Gite et al.^28^	U-Net++ for lung segmentation	Enhances TB detection with advanced segmentation	138 X-ray images	IoU: 0.95	Specific to X-ray images, not other modalities
Arvind et al.^29^	Modified U-Net with dropouts	Reduces overfitting, enhances model generalization	900 X-ray images	Accuracy: 93.87%	Optimized for lung segmentation, less versatile for other organs
Chen et al.^30^	LDANet combines RSA and GCA	Enhances lung CT segmentation via dual attention	LIDC-IDRI	Dice similarity coefficient (DSC): 98.430%	Sensitive to variations in imaging protocols
Ghali et al.^31^	Dual loss functions with five models	Optimizes CXR segmentation for various lung conditions	662 X-ray images	F1 score: 97.47%	May not generalize across all imaging equipment types
Murugappan et al.^32^	DeepLabV3 + with various networks	Enhances segmentation of lung and infected areas	750 CT images	IoU: 0.9971	Performance varies with different network layers
Ambesange et al.^33^	FL with U-net and transfer learning	Enhances lung X-ray segmentation while preserving data privacy	662 X-ray images	Accuracy: 98.92%	Limited by data variability across nodes
Hasan et al.^34^	Deeplabv3plus with Atrous Convolution	Optimizes feature resolution for segmentation	558 X-ray images	Accuracy: 97.42%	May struggle with highly variable image quality
Swaminathan et al.^35^	Wiener filter, GAN with SSSOA, VGG16 classification	Streamlines detection process with an integrated model	LIDC-IDRI	Accuracy: 97%	Relies on high-quality pre-processed images
Suji et al.^36^	U-Net with EfficientNet-b3, transfer learning	Optimizes nodule segmentation with transfer learning	LIDC-IDRI	IoU: 0.45	Performance variability across datasets
Thangavel and Palanichamy^37^	T-Net, CenterNet, NASNet with preprocessing	Automates nodule classification efficiently	LIDC-IDRI	DSC: 99.07%	May require fine-tuning for new datasets
Cai et al.^38^	GANs for image translation segmentation	Enhances lung CT segmentation with GANs	267 CT images	Accuracy: 89.63%	May not handle all types of lung anomalies
Ramos and Pineda^38^	Tri-phase semi-automated segmentation with preprocessing	Provides consistent, swift segmentation results	267 CT images	IoU: 0.9341	May require manual adjustments for optimal results
Zheng et al.^40^	Threshold-gradient with TFDM and convex hull repair	Enhances segmentation performance and robustness	2112 CT images	IOU: 0.9911	Requires precise calibration of threshold settings
Guo et al.^41^	whale optimization algorithm-based random mutation strategy for image segmentation	Speeds up convergence, improves segmentation	25,000 CT images	Feature similarity index measure (FSIM): 81.57%	May not adapt well to other cancer types
Pandey and Bhandari^42^	Morphological filtering and SVM classification	Enhances tumor visibility for early intervention	LIDC-IDRI	Accuracy: 87.79%	May miss micro-tumors or highly diffuse anomalies
Vijayakumar et al.^43^	CapsNet with RGF and SMFMF, U-net segmentation	Enhances lung cancer detection efficiency	1097 CT images	Accuracy: 98%	May underperform with non-standardized data
Qiao et al.^44^	WSTSA with genetic algorithm	Streamlines optimal CNN architecture selection	2773 CT images	DSC: 72.40%	Dependent on initial algorithm parameters
Li et al.^45^	Attention and Sobel edge detection	Enhances lesion feature extraction and performance	LIDC-IDRI	Accuracy: 93.48%	May require high computational resources
Murugaraj et al.^46^	HM-LeNet with NLM filter and K-Net	Classifies lung abnormalities efficiently	23 CT scans images	Accuracy: 92.7%	Potential overfitting to specific abnormalities
Fu et al.^47^	LDN-SNP with SNP-type neurons	Efficient, compact segmentation of COVID-19 CT	100 CT scans images	Accuracy: 97.0% and DSC: 74.0%	Limited adaptability to other imaging tasks
Erciyes et al.^48^	ANN with optimized U-Net and augmentation	Reduced overfitting with enhanced segmentation	American association of physicists in medicine (AAPM) thoracic auto-segmentation	DSC: 94.68% and IoU: 0.8990	Limited adaptability to unseen pathologies
Yang et al.^49^	3D model with focal and dice loss	Improved deep and shallow feature representation	199 CT scans images	Accuracy: 99.90% and DSC: 56.10%	Overfitting risk from a small COVID dataset
Nguyen et al.^50^	Two-stage no-new-U-Net with attention and boundary loss	Improved GGO segmentation with fuzzy boundary handling	Post-COVID CT challenge	DSC: 71.93%	Difficulty generalizing to non-GGO lung lesions
Vincy et al.^51^	3D ResNet50 encoder with dense-feature U-Net decoder	Enhanced multiscale tumor features from lung CT slices	LUNA16	IoU: 0.738	Contrast issues in small or diffuse tumors
Jannat et al.^52^	Lightweight residual U-Net with CBAM and ASPP	Improved lung mask extraction	Japanese society of radiological technology (JSRT)	DSC: 98.72%	Limited generalization on low-quality images

### Tumor detection

In 2023, Vijh et al.^53^ presented a hybrid bio-inspired algorithm combining the WOA with adaptive particle swarm optimization (APSO) to enhance feature selection in CNNs, aiming to heighten the performance of tumor detection in lung CT scans. Despite its innovative integration, one shortfall may be the potential for increased computational complexity that hybrid algorithms often introduce. Alshayeji and Abed^54^ developed a machine-learning model based on extreme gradient boosting (XGBoost) to classify lung cancer CT images and identify nodule malignancy stages. This framework combines conventional machine learning with automatic segmentation, using DeepLabv3 + initialized with ResNet-18 for precise nodule segmentation. This comprehensive CAD system offers practical support for radiologists by facilitating accurate malignancy-stage identification and nodule segmentation. Halder and Dey^55^ introduced a framework that uses atrous convolution for segmenting and characterizing lung nodules from high-resolution computed tomography (HRCT) images. The proposed model, featuring a two-layer atrous pyramid and residual connections, captures multi-scale features to improve lung nodule detection and classification.

In 2024, Venkatesh et al.^56^ proposed a technique using patch processing alongside deep learning to classify lung tumors in CT images swiftly and efficiently. However, patch processing might miss contextual information from larger image areas, possibly affecting the robustness of the classification. Sundarrajan et al.^57^ developed a cutting-edge method for lung tumor detection that employs the cloud-based Internet of things (IoT) for data aggregation and an optimized fuzzy C-means neural network (OFCMNN) for segmentation, complemented by a kernel multilayer deep transfer convolutional learning (KM-DTCL) ensemble. Despite its sophistication, reliance on cloud-based IoT could raise concerns about data security and latency issues. Srinivasulu et al.^58^ used autoencoders to detect lung malignancies in CT images, achieving high performance and refined feature extraction. Yet, the complexity of the model could hinder real-world applicability where computational resources are limited. Tang and Zhang^59^ engineered a multi-functional network incorporating a unified base architecture and prediction refinement framework to enhance the segmentation and identification of lung nodules. This network features a coarse segmentation subnetwork (Coarse Seg-net) and a fine segmentation subnetwork (Fine Seg-net) alongside a cooperative classification subnetwork (Class-net). The Coarse Seg-net performs initial localization, which improves the Fine Seg-net and Class-net by leveraging task correlations for better segmentation and classification. Kongkham et al.^60^ explored deep-learning applications for the segmentation of lung nodules using CT scans. The study harnesses CNNs to overcome the limitations of conventional segmentation methods such as thresholding and region-growing. By utilizing CNNs, this research significantly enhances the detection and delineation of lung nodules, capitalizing on their ability to learn complex patterns from extensive data. Gautam et al.^61^ developed a cutting-edge ensemble of deep-learning models to improve the classification of lung nodule severity from CT images. This approach harnesses the capabilities of three advanced convolutional neural networks: ResNet-152, DenseNet-169, and EfficientNet-B7, integrating them through a novel ensemble method that uses a weight optimization strategy based on receiver operating characteristic area under the curve (ROC-AUC) and F1-scores. Sadremomtaz and Zadnorouzi^62^ introduced an enhanced U-Net neural network for precisely segmenting pulmonary nodules in CT scans. The model improves feature extraction and network efficiency by integrating dilation residual structures and spatial-channel attention mechanisms, making it robust for diverse nodule characteristics. Pan et al.^63^ introduced a semi-supervised training strategy called entropy minimization lesion-level data augmentation, which uses a novel augmentation technique to utilize limited labeled CT data. The data augmentation method accelerates feature learning for lesion contours, while entropy minimization reduces overfitting and improves prediction confidence. Rathan and Lokesh^64^ developed a hybrid lung cancer detection and staging system that integrates deep learning techniques with active contour models for segmentation and a high-resolution network (HRNet) for stage classification. This comprehensive approach targets both the detection of pulmonary disease and the precise classification of lung cancer stages, enhancing diagnostic reliability.

In 2025, Shi and Zhang^65^ developed an enhanced U-Net framework to tackle the challenges of segmenting small and ambiguously defined pulmonary nodules. The model incorporates three innovative modules: the locality-aware attention module, which intensifies the focus on areas containing nodules; the pixel transformer module, which refines semantic information processing; and the perceptual adaptation module, which adjusts to the scale of the receptive field effectively. This comprehensive approach aims to improve the effectiveness of early lung cancer diagnosis by precisely segmenting pulmonary nodules. Miao et al.^66^ developed a multimodal deep learning nomogram (DLN) that utilizes features from pulmonary nodules and intrathoracic fat (ITF) to differentiate between benign and malignant nodules. The method incorporates precise intranodular and perinodular regions (IPN) segmentation, feature extraction via the Swin transformer, and feature selection using least absolute shrinkage and selection operator (LASSO) regression^67^. Yu et al.^68^ proposed a two-step multimodal neural architecture search framework for optimizing lung nodule classification using positron emission tomography (PET)/CT data. The method combines a sandwich rule and an in-place distillation strategy for training, followed by a parallel operation edge decomposition strategy to further refine performance. Jiang et al.^69^ proposed a framework for pulmonary nodule detection in CT images that integrates neural network architectures with specialized attention mechanisms. This framework includes a partial attention module based on Kolmogorov–Arnold networks (KAN), which enhances global representation learning within a conventional CNN. Additionally, the model incorporates an adaptive feature fusion module and a multi-slice partition channel priority attention module, specifically designed to improve the detection of small nodules by better handling small-scale features. Xue et al.^70^ introduced a novel deep learning model called the squeeze-and-excitation vision transformer (SE-ViT), designed to enhance the interpretability and effectiveness of lung nodule classification. The SE-ViT integrates two attention mechanisms: self-attention for capturing relationships between different image patches and the SE mechanism to prioritize crucial information within those patches. This model addresses the challenges of lung nodule variability in shape and texture by focusing on the essential features that define malignancy, providing visual interpretations through gradient-weighted class activation mapping (Grad-CAM)-generated attention maps to aid clinical decision-making. Elhassan et al.^71^ proposed a dual-model framework that integrates a CNN for lung tissue classification and the You Only Look Once version 8 (YOLOv8) for real-time tumor localization, supported by a deep convolutional GAN (DCGAN)-based data augmentation to enhance model robustness. Abdulqader et al.^72^ introduced a deep learning framework that combines transformer-based attention layers, adaptive anchor-free detection, and an enhanced feature pyramid network (FPN). The model was trained with a multi-task loss function to optimize the detection, classification, and localization of lung tumors in CT scans. Liu et al.^73^ proposed a lung nodule detection model based on YOLOv11. The model integrates MobileNetV4, multi-scale dilation attention (MSDA), and frequency fusion bidirectional FPN, along with novel loss functions to enhance feature fusion and prediction quality. Li^74^ developed a lung nodule detection framework that combines the global channel spatial attention mechanism (GCSAM), candidate nodule detection network (CNDNet) with a Res2Net backbone, and a false positive reduction network (FPRNet). The framework is further enhanced by hierarchical progressive feature fusion (HPFF) to support robust multi-scale nodule representation.

Table 3 offers an exhaustive analysis of the most advanced models for detecting tumors. Many previous studies rely on standard loss configurations, mainly classification loss^53–55^ and bounding box regression loss^61,71–74^, to separate tumor regions from non-tumor tissues. Although effective in many cases, these methods often struggle in low-contrast regions or where tumor boundaries are vague. For instance, the models by Vijh et al.^53^ and Gautam et al.^61^ employ CNN-based or ensemble classification strategies but do not address fine-grained structural accuracy. Similarly, segmentation-oriented studies, such as those by Halder and Dey^55^ and Sadremomtaz and Zadnorouzi^62^, focus on multiscale feature extraction and attention mechanisms. However, they do not use adversarial techniques to ensure structurally realistic segmentation outputs. These limitations often result in increased false positives or false negatives, particularly in areas of ambiguity.

To address these challenges, our method uses a composite loss function within a specialized GAN architecture. This loss function combines classification loss, bounding box regression loss, and a new adversarial loss component. Unlike conventional loss functions, the adversarial component regularizes tumor structure by learning from differences between predicted and ground-truth tumor regions at the feature level. This reduces detection errors and improves diagnostic confidence.

**Table 3:** Comparison of advanced models for tumor detection.

Authors	Methodology	Contribution	Dataset	Result	Limitation
Vijh et al.^53^	Hybrid WOA and APSO with CNN	Optimizes feature selection for tumor classification	120 CT images	Accuracy: 97.18%	May require extensive parameter tuning
Alshayeji and Abed^54^	XGBoost and DeepLabv3+	Integrates machine learning and advanced segmentation for comprehensive nodule analysis	LIDC-IDRI	Accuracy: 99.65%	Requires detailed nodule characteristics
Halder and Dey^55^	Atrous pyramid and residual connections	Applies atrous convolution for detailed multi-scale feature extraction	LIDC-IDRI	Accuracy: 95.97% and DSC: 97.15%	May perform variably across different CT scanners
Venkatesh et al.^56^	Patch processing with deep learning	Introduces patch-based preprocessing for enhanced classification	LIDC-IDRI	Accuracy: 95.70%	Relies on high-quality CT image preprocessing
Sundarrajan et al.^57^	Cloud-IoT with OFCMNN and KM-DTCL	Integrates cloud-IoT for data collection and segmentation-classification pipeline	LIDC-IDRI	Accuracy: 93.86%	Dependence on cloud-IoT infrastructure
Srinivasulu et al.^58^	Autoencoder	Integrates advanced neural network structures for nuanced feature extraction in lung tumor detection	Open-source and online portals	Accuracy: 96%	May not generalize well to less curated, real-world clinical data
Tang and Zhang^59^	Shared backbone and distillation	Leverages task correlations for enhanced performance	LIDC-IDRI	Accuracy: 91.9%	Require careful tuning of task interdependencies
Kongkham et al.^60^	CNNs for lung nodule segmentation	Introduces advanced CNN techniques to refine nodule segmentation	LIDC-IDRI	DSC: 85.3%	Requires large annotated datasets for optimal training
Gautam et al.^61^	ResNet-152, DenseNet-169, EfficientNet-B7 ensemble	Enhances nodule classification through a weighted ensemble approach	LIDC-IDRI	Accuracy: 97.23%	Requires sophisticated model integration skills
Sadremomtaz and Zadnorouzi^62^	Enhanced U-Net with dilation residuals and attention mechanisms	Enhances feature extraction and network efficiency	LIDC-IDRI	Accuracy: 99.92%	Initial model setup and training can be computationally intensive
Pan et al.^63^	semi-supervised training strategy for entropy minimization lesion-level data augmentation	Enhances training efficiency with novel data augmentation	LIDC-IDRI	DSC: 81.06%	Requires careful balancing in parameter tuning
Rathan and Lokesh^64^	Active contour & HRNet for lung cancer	Utilizes hybrid imaging techniques for nuanced disease staging	LIDC-IDRI	Accuracy: 98.4%	Requires high-quality imaging for optimal performance
Shi and Zhang^65^	Advanced UNet with attention and adaptation	Tailors segmentation sensitivity to nodule characteristics	LIDC-IDRI	DSC: 89.85% and IOU: 0.8960	Requires calibration for variable nodule sizes
Miao et al.^66^	DLN with ITF and IPN integration	Integrates ITF features to enhance nodule characterization	1,385 CT images	Accuracy: 82.3%	Dependent on the quality of fat and nodule imaging
Yu et al.^68^	Two-step multimodal one-shot neural architecture search for PET/CT pulmonary nodules	Introduces a structured two-step neural architecture search to optimize architecture search	499 CT images	Accuracy: 94.23%	Intensive computational resources required
Jiang et al.^69^	KAN and adaptive feature fusion	Enhances nodule detection by optimizing feature representation	Lung nodule analysis 2016 (LUNA16)	Sensitivity: 94.73%	Require extensive training data for optimization
Xue et al.^70^	SE-ViT with dual attention	Integrates self-attention and SE mechanisms for detailed feature analysis	LUNA16	Accuracy: 86.3%	High dependency on the quality of training data
Elhassan et al.^71^	CNN + YOLOv8 with DCGAN augmentation	Enhanced real-time tumor detection and classification	Iraq‑oncology teaching hospital /national center for cancer diseases (IQ-OTHNCCD)	IOU: 0.85	Sensitivity to imaging conditions and variations
Abdulqader et al.^72^	Transformer, anchor-free, FPN with multi-task learning	Unified detection, classification, and localization of lung tumors	1608 CT images	IoU: 0.9576	Complex training pipeline for clinical deployment
Liu et al.^73^	YOLOv11 with MobileNetV4, MSDA, frequency fusion bidirectional FPN, slide loss	Improved detection via multi-scale attention and feature integration	LUNA16	IoU: 0.946	High architectural complexity for deployment
Li^74^	CNDNet + FPRNet with GCSAM and HPFF	Enhanced multi-scale detection and false positive reduction	LUNA16	Sensitivity: 97.7%	May misclassify nodules with unclear margins

### 3D tumor reconstruction

Recent medical progress has spurred the advancement of 3D models, greatly improving patient treatment approaches^75–79^. Liver resection surgeries, for instance, benefit significantly from 3D models. These models offer surgeons detailed insights into liver anatomy, allowing for more precise preoperative planning and intraoperative guidance. They can also aid in identifying anatomical variations and potential complications, improving surgical outcomes^80,81^. Additionally, research has explored generating 3D brain models from MRI data^82^.

A significant focus in current medical research is on reconstructing 3D models of lung tumors^83^. Hong et al.^10^, Gu et al.^83^, and Rezaei et al.^84^ employed a GAN-based approach for 3D image reconstruction. Their approach involved snake optimization, a technique that uses an initial contour to iteratively refine the boundary of an object for lung segmentation. They also used Gustafson and Kessel (GK) clustering, a method for grouping similar data points into clusters, for tumor segmentation. Nonetheless, the performance of snake optimization is critically reliant on the positioning of the initial contour^85^ since incorrect initial placements can result in segmentation inaccuracies. Snake optimization faces challenges with intricate lung structures, such as atypical shapes or nodules, which hampers its ability to accurately delineate boundaries. Furthermore, the performance of the GK clustering relies on selecting optimal initial centroids, affecting tumor segmentation quality. Challenges arise with overlapping tumors or heterogeneous characteristics, complicating accurate data point assignment to clusters^83^. Karrar et al.^86^ developed a method for 3D reconstruction of lung nodules from 2D CT scans to assist in early treatment decisions. Their technique involves a segmentation approach using bounding boxes and maximum intensity projection to extract lung nodules from candidate masses. A rule-based classifier, an algorithm that makes decisions based on predefined rules, selects the relevant nodules for 3D reconstruction. These nodules are then visualized through 3D Slicer (version 7) software using surface rendering techniques. However, the method may be limited by the effectiveness of rule-based classifiers and the quality of initial 2D scans, which can affect the reliability of nodule extraction and subsequent 3D modeling. Dlamini et al.^87^ presented a framework for identifying, segmenting, and reconstructing non-small cell lung cancer tumors utilizing YOLOv4 and an active contour method. The framework is divided into two primary components: the detection and volumetric visualization phases. Detection involves image enhancement to reduce noise, augmentation to diversify the dataset, and labeling for accurate localization. In the volumetric rendering section, images undergo filtering, tumor extraction through clustering, and region-based active contour modeling for 3D reconstruction. This methodology utilizes enhanced images and precise contour data for effective tumor rendering. Challenges here include potential overfitting due to augmented data variability and the dependency on the effectiveness of the YOLOv4 model in varying imaging conditions. Shi et al.^88^ developed a novel model utilizing ViT blocks to reconstruct 3D lung volumes from single 2D chest x-ray images. This model uses global information and long-range relationships to improve 3D reconstruction. It incorporates a pooling layer to refine feature extraction and employs patch discriminators to generate smoother and more realistic 3D lung models. Additionally, subject demographics are used as auxiliary inputs to refine reconstruction performance. This approach merges traditional imaging data with demographic insights, enhancing the fidelity of the output. Najafi et al.^2^ and Huang et al.^89^ proposed a three-step framework that combines GAN, LSTM, and a VGG16. Two customized GANs perform lung and tumor segmentation, followed by VGG16-LSTM feature extraction and final 3D tumor reconstruction.

Table 4 presents an exhaustive analysis of the most advanced models for the 3D reconstruction of lung tumors. Several studies, such as those by Hong et al.^10^ and Karrar et al.^86^, rely on statistical modeling techniques or rule-based bounding box extraction, which often limit the flexibility of reconstruction when dealing with irregular or noisy tumor structures. In contrast, deep learning-based approaches, such as Najafi et al.^2^ and Huang et al.^89^, utilize LSTM for sequence modeling and feature transfer. However, these methods are limited in capturing localized data variations because standard LSTM architectures rely on generalized temporal patterns learned from the full training dataset. This global modeling may not generalize well when reconstructing fine-grained, patient-specific tumor boundaries.

To overcome these challenges, our model introduces a hybrid GAN and TLSTM architecture, where the TLSTM component adjusts its weight assignment based on the proximity of data points to the target rather than relying solely on global sequence trends. This localized adaptation enhances model sensitivity in the edge regions of tumors, improving reconstruction fidelity, particularly in complex or atypical cases. By incorporating adversarial learning alongside transductive temporal modeling, the proposed method provides both structural realism and data-specific precision, distinguishing it from prior models.

**Table 4:** Comparison of advanced models for the 3D reconstruction of lung tumors.

Authors	Methodology	Contribution	Dataset	Result	Limitation
Hong et al.^10^	VGG and GK clustering	Introduces GAN-based 3D reconstruction for lung tumor imaging	LUNA16	HD: 2.82 and ED: 2.82	Performance may vary for non-lung tumor datasets
Gu et al.^83^	ResNet and GK clustering	Combines GAN and LSTM for feature-driven 3D tumor reconstruction	LUNA16	HD: 2.99 and ED: 1.06	Requires well-segmented 2D input images
Rezaei et al.^84^	VGG and GK clustering	Uses GAN for 3D reconstruction and transfers 2D image features	LUNA16	HD: 3.02 and ED: 1.06	Relies heavily on accurate segmentation in preprocessing
Karrar et al.^86^	3D reconstruction using bounding boxes and rule-based classifier	Develops an approach for nodule extraction and surface rendering for 3D modeling	LIDC-IDRI	Accuracy: 99.6627%	Limited robustness to nodules with irregular shapes
Dlamini et al.^87^	YOLOv4 and region-based active contour model	Integrates detection and volumetric rendering in a single automated pipeline	LIDC-IDRI	Accuracy: 99.74% and DSC: 92.19%	Requires robust preprocessing for noise removal
Shi et al.^88^	ViT and demographics	Enhances 3D reconstruction from 2D images using ViT	2525 chest x-rays	DSC: 76.9%	Dependent on the inclusion of accurate demographic data
Najafi et al.^2^	GAN, LSTM, and VGG16	Integrates GANs with VGG16 and LSTM for accurate 3D reconstruction	LIDC-IDRI	HD: 0.986 and ED: 1.126	Training instability due to sensitivity to input variations and multi-model tuning
Huang et al.^89^	GAN, attention-based LSTM, and VGG16	Integrates multi-stage GANs with VGG16 and attention-based LSTM for accurate 3D reconstruction	LIDC-IDRI	HD: 2.963 and ED: 1.725	Requires extensive labelled data and balanced segmentation to maintain accuracy across GAN stages

## Background

This section briefly overviews TLSTM, spatial attention, RL, trust region policy optimization (TRPO), and PPO.

### TLSTM

LSTM is designed to handle the shortcomings of traditional recurrent neural networks (RNNs), precisely their inability to learn long-range dependencies in sequence data^90,91^. An LSTM is structured to remember information for extended periods, which is crucial in tasks like language modeling and time series prediction^92^.

**Fig. 1:**
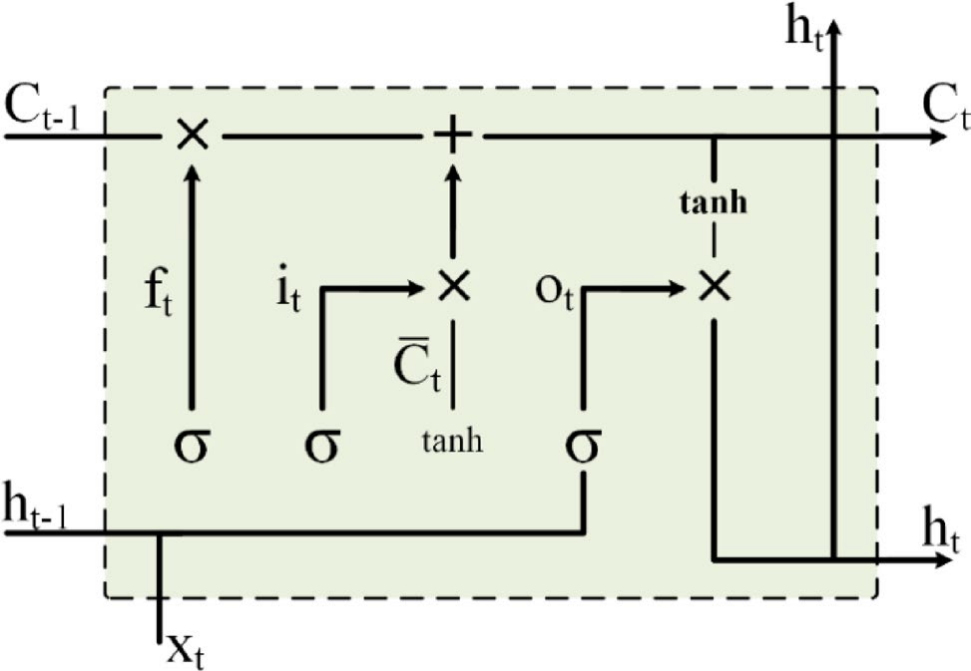
Architecture of an LSTM unit. It displays the key components, including the input gate ($\:{i}_{t}$), forget gate ($\:{f}_{t}$), output gate ($\:{o}_{t}$), memory cell ($\:{c}_{t}$), and hidden state ($\:{h}_{t}$), which collectively manage the flow of information over time, ensuring data is appropriately stored, retained, and retrieved.

The LSTM model^93^ uses a gating mechanism to control information flow. Figure 1 illustrates the structure of an LSTM unit, which oversees the storage, retention, and retrieval of data from the memory cell over time. LSTMs feature three unique gates for effective information processing. The various components of an LSTM, including the input gate ($\:{i}_{t}$), forget gate ($\:{f}_{t}$), output gate ($\:{o}_{t}$), memory cell ($\:{c}_{t}$), and hidden state ($\:{h}_{t}$) for a specific time t and input $\:{x}_{t}$, are defined by Eqs. 1–5^14^:

1 $$\:{i}_{t}=\sigma\:({W}_{xi}{x}_{t}+\:\:{W}_{hi}{h}_{t-1}+{W}_{ci}{c}_{t-1}+\:{b}_{i})$$

2 $$\:{f}_{t}=\sigma\:({W}_{xf}{x}_{t}+\:\:{W}_{hf}{h}_{t-1}+{W}_{cf}{c}_{t-1}+\:{b}_{f})$$

3 $$\:{c}_{t}={f}_{t}{c}_{t-1}+{i}_{t}tanh({W}_{xc}{x}_{t}+\:\:{W}_{hc}{h}_{t-1}+\:{b}_{c})$$

4 $$\:{o}_{t}=\sigma\:({W}_{xo}{x}_{t}+\:\:{W}_{ho}{h}_{t-1}+{W}_{co}{c}_{t}+\:{b}_{o})$$

5 $$\:{h}_{t}={o}_{t}tanh\left({c}_{t}\right)$$

Here, $\:\sigma\:(.)$ serves as the sigmoid activation function of the gate and can take the form of either a logistic sigmoid or a hyperbolic tangent function. The hyperbolic tangent, denoted by $\:tanh$, normalizes the output within the range of -1 to 1. $\:{W}_{xk}$, where $\:k\in\:\{i,\:f,\:o,\:c\}$, is associated with the input, forget, output gates, and the cell state, respectively. Similarly, $\:{W}_{hk}$, where $\:\text{k}\in\:\{i,\:f,\:o,\:c\}$, denotes the weights connected to the previous hidden state $\:{h}_{t-1}$. Moreover, $\:{W}_{ck}$, where $\:k\in\:\{i,\:f,\:o\}$, represents the diagonal weight arrays that link the cell state to these respective gates. Bias values for the gates and the memory cell equations are indicated as $\:{b}_{i}$, $\:{b}_{f}$, $\:{b}_{c}$, and $\:{b}_{o}$, respectively. The LSTM parameters, including weights and biases, are denoted as column vectors $\:{w}_{lstm}$ and $\:{b}_{lstm}$. The overall operation of the LSTM is summarized in Eq. 6, where the functions $\:g( \cdot )$ and $\:f( \cdot )$ are based on Eqs. 1–5^14^.

6 $$\:\left\{\begin{array}{c}{c}_{t}=f({c}_{t-1},{h}_{t-1},{x}_{t};{w}_{lstm},{b}_{lstm})\:\\\:{h}_{t}=g({h}_{t-1},{c}_{t-1},{x}_{t};{w}_{lstm},{b}_{lstm})\end{array}\right.$$

After the debut of the LSTM model, numerous advancements and variations, such as TLSTM, have been developed to enhance its performance across various studies. The state space of TLSTM is defined in Eq. 7^14^:

7 $$\:\left\{\begin{array}{c}{c}_{t,\eta\:}=f({c}_{t-1,\eta\:},{h}_{t-1,\eta\:},{x}_{t};{w}_{lstm,\eta\:},{b}_{lstm,\eta\:})\:\\\:{h}_{t,\eta\:}=g({h}_{t-1,\eta\:},{c}_{t-1,\eta\:},{x}_{t};{w}_{lstm,\eta\:},{b}_{lstm,\eta\:}\end{array}\right.$$

In this context, η represents an unseen sequence. The formulation of Eq. 7 significantly differs from that of Eq. 6, as the parameters of the model adjust based on the characteristics of the evaluation point. This adaptation reflects the modifications introduced by new data, represented by $\:\eta\:$ and denoted as $\:z\left(\eta\:\right)$.

#### Spatial attention

Spatial attention allows TLSTM to selectively concentrate on particular input data segments simultaneously instead of uniformly processing the entire dataset. It highlights essential variables and attributes, protecting the model from irrelevant data or noise^94^.

Spatial attention is typically implemented as an intermediate layer within the TLSTM architecture, positioned between the input and the TLSTM layers. It aggregates the input data in a weighted manner, where the weights are assigned during training according to the significance of each input feature. The input to this spatial attention layer is an $\:N\times\:D$ matrix $\:X$, where $\:N\:$represents the number of temporal instances, and $\:D$ represents the number of features. The spatial attention mechanism assigns weights based on the input data and the parameters learned during training, represented by $\:W$ and$\:\:b$. These weight assignments are specified in Eq. 8:

8 $$\:a=softmax\:(XW+b)$$

The subsequent step is the computation of the weighted input sum as described in Eq. 9:

9 $$\:{X}^{{\prime\:}}=Xa$$

This weighted sum condenses the input while steering the attention of the model toward the most impactful features.

Spatial attention boosts the adaptability of TLSTM by selectively emphasizing critical features. It minimizes the likelihood of overfitting by curbing excessive focus on non-essential noise or details, which could distort the learning outcomes. Moreover, this focused strategy aids in managing high-dimensional data by reducing the computational load associated with processing less critical information. Consequently, a TLSTM outfitted with spatial attention excels at processing varied and intricate datasets, yielding more uniform and widely applicable results. Such enhancements in model performance are especially vital in situations where detecting subtle data variations is key to making accurate predictions.

### RL

RL represents a process where an agent enhances its decision-making abilities by undertaking actions that amplify the rewards obtained from its environment. This evolving procedure is configured utilizing Markov decision processes (MDPs). An MDP is characterized by elements $\:(S,\:A,\:P,\:{\rho\:}_{0},\:r)$, where $\:S$ embodies the complete set of potential states, and $\:A$ includes all feasible actions. The function $\:P:\:S\:\times\:\:A\:\times\:\:S\:\to\:\:R$ calculates the likelihood of transitioning from one state to another based on the selected action. The function $\:{\rho\:}_{0}$: $\:S\:\to\:\:R$ represents the initial state probability distribution, and $\:r:\:S\:\times\:\:A\:\to\:\:R$ provides the reward feedback for each state-action pair.

In RL, at each timestep $\:t$, an observer assesses the present state $\:{s}_{t}$​ and decides on an action $\:{a}_{t}$​ guided by a strategy $\:\pi\::\:S\:\times\:\:A\:\to\:\:\left[\text{0,1}\right]$. After executing this action, the environment rewards the decision-maker with $\:r({s}_{t},{a}_{t})$ and transitions to the next state $\:{s}_{t+1}$. Throughout this sequence, the observer calculates the aggregate discounted reward starting from timestep $\:t$, represented as $\:{R}_{t}=\sum\:_{t=0}^{{\infty\:}}{\gamma\:}^{t}r({s}_{t},{a}_{t})$, where $\:\gamma\:$ is the discount factor that values future rewards more heavily. Based on this return, the value functions for the state $\:{V}_{\pi\:}\left({s}_{t}\right)$, the action $\:{Q}_{\pi\:}\left({s}_{t},{a}_{t}\right)$ at the state-action pair, and the advantage $\:{A}_{\pi\:}\left({s}_{t},{a}_{t}\right)$ are determined as follows:

10 $$\:{V}_{\pi\:}\left({s}_{t}\right)={E}_{{a}_{t},{s}_{t+1},\dots\:\sim\pi\:\:}\left[\sum\:_{k=t}^{\infty\:}{\gamma\:}^{k-t}r({s}_{k},{a}_{k})\right]$$

11 $$\:{Q}_{\pi\:}\left({s}_{t},{a}_{t}\right)={E}_{{s}_{t+1},{a}_{t+1},\dots\:\sim\pi\:\:}\left[\sum\:_{k=t}^{\infty\:}{\gamma\:}^{k-t}r({s}_{k},{a}_{k})\right]$$

12 $$\:{A}_{\pi\:}\left({s}_{t},{a}_{t}\right)={Q}_{\pi\:}\left({s}_{t},{a}_{t}\right)-{V}_{\pi\:}\left({s}_{t}\right)$$

The goal is to formulate a policy, $\:\pi\:$, that maximizes the cumulative discounted rewards from the initial state, as detailed below^95^:

13 $$\:\eta\:\left(\pi\:\right)={E}_{{s}_{0},{a}_{0},\dots\:}\left[{R}_{0}\right]={E}_{{s}_{0},{a}_{0},\dots\:}\left[\sum\:_{t=0}^{\infty\:}{\gamma\:}^{t}r({s}_{t},{a}_{t})\right]\:$$

#### TRPO

To enhance the performance objective outlined in Eq. 13, TRPO modifies strategies using available policy data to optimize a different objective, ensuring the Kullback-Leibler (KL) divergence remains below a predetermined limit, thus keeping policy changes restrained^96^:

14 $$\:\underset{\pi\:}{\text{max}}{E}_{s\sim{\rho\:}_{{\pi\:}_{{\theta\:}_{old}}},\:\:a\:\in\:\:{\pi\:}_{{\pi\:}_{old}}}\left[\frac{{\pi\:}_{\theta\:}\left(a|s\right)}{{\pi\:}_{{\theta\:}_{old}}\left(a|s\right)}{A}_{{\pi\:}_{old}}\left(s,a\right)\right]\:$$

subject to

15 $$\:{E}_{s\sim{\rho\:}_{{\pi\:}_{old}}}\left[{D}_{KL}\left({\pi\:}_{old}\right(.\left|s\right)\left|\right|\:\pi\:(.|s\left)\right)\right]\le\:\delta\:\:$$

Here, $\:{\pi\:}_{old}$ denotes the previous policy, and $\:\delta\:$ represents the allowable divergence limit. The expression $\:{D}_{KL}\left({\pi\:}_{old}\right(.\left|s\right)\left|\right|\:\pi\:(.|s\left)\right)$ calculates the KL divergence, reflecting the extent to which the novel policy $\:\pi\:$ deviates from the earlier policy $\:{\pi\:}_{old}$ for a given state $\:s$. The variable $\:{\rho\:}_{{\pi\:}_{old}}$ signifies the discounted state distribution originating from the initial state $\:{s}_{0}$ under the prior policy $\:{\pi\:}_{old}$, formulated as $\:{\rho\:}_{{\pi\:}_{old}}\left(s\right)=\sum\:_{t=0}^{{\infty\:}}{\gamma\:}^{t}P({s}_{t}=s|{s}_{0},{\pi\:}_{old})$. Targeting the alternate objective without restraining the KL divergence might induce drastic, unforeseen changes in the policy.

#### PPO

To reduce significant policy shifts, PPO utilizes an adapted optimization target termed the clipped surrogate objective, arranged as described below^73^:

16 $$\:L_{{PPO}}^{{CLIP}} = \:E_{{s\sim \rho \:_{{\pi {\kern 1pt} _{{old}} ,{\kern 1pt} {\kern 1pt} {\kern 1pt} a\sim \pi {\kern 1pt} _{{old}} }} }} \left[ {{\text{min}}\left( {\frac{{\pi \:\:\left( {a|s} \right)}}{{\pi \:_{{old}} \left( {a|s} \right)}}A_{{\pi \:_{{old}} }} \left( {s,a} \right),clip\left( {\frac{{\pi \:\left( {a|s} \right)}}{{\pi \:_{{old}} \left( {a|s} \right)}},1 - \in \:,1 + \in \:} \right)A_{{\pi \:_{{old}} }} \left( {s,a} \right)} \right)} \right]$$

where $\:\epsilon\:$, a small positive scalar, is set to safeguard policy stability while experimenting with novel actions. The advantage function $\:{A}_{{\pi\:}_{old}}\left(s,a\right)$ appraises the efficacy of an action $\:a$ at state $\:s$ under the prior policy $\:{\pi\:}_{old}$, relative to the expected results from alternative actions at the state. This function assesses whether an action outperforms or underperforms the typical expectations under the existing policy. In this methodology, clipping curtails excessive alterations to the policy during training, promoting gradual and reliable enhancements. The specifics of this clipping are outlined below^97^:

17 $$\:clip(x,\:a,\:b)\:=\:max(a,\:min(b,\:x\left)\right)$$

In this context, $\:x$ is the variable being adjusted, with $\:a$ defining the minimum limit and $\:b$ marking the maximum limit of the interval restricting $\:x$. The surrogate objective, detailed in Eq. 16, aims to curb substantial policy alterations by imposing penalties on changes that drastically shift $\:\frac{\pi\:\left(a|s\right)}{{\pi\:}_{old}\left(a|s\right)}$ away from 1. However, a significant limitation of PPO is its heavy reliance on on-policy data, which makes the data collection process more challenging because it integrates only a small portion of off-policy data, requiring frequent interactions between the agent and its environment.

## Materials and methods

**Fig. 2:**
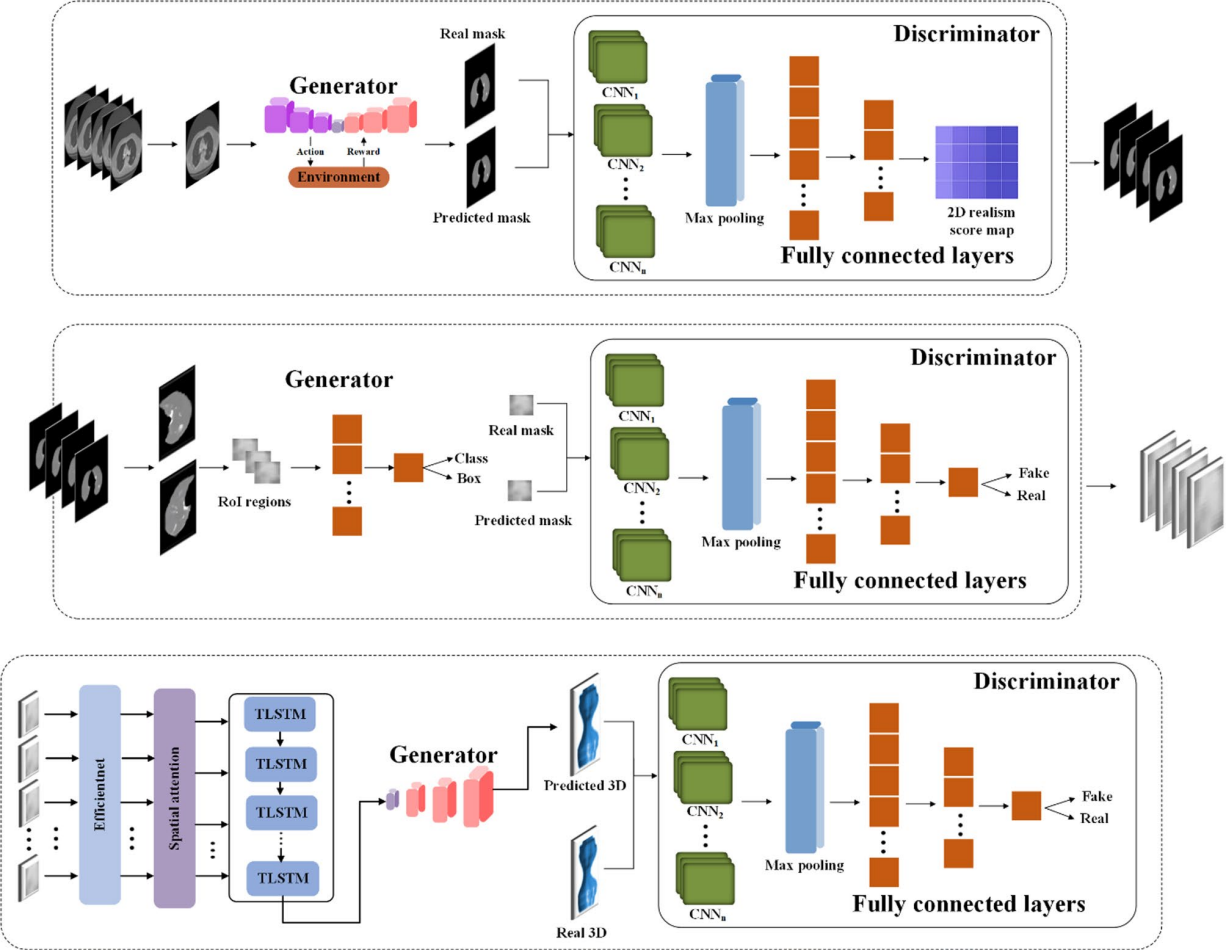
Comprehensive framework of the proposed model. The figure demonstrates the integrated process encompassing lung segmentation, tumor detection, and 3D tumor reconstruction. The lung segmentation phase employs GAN and U-Net architectures, utilizing off-policy PPO to address imbalanced classification. The tumor detection model incorporates a GAN alongside Mask R-CNN, enhanced by a novel loss function for more accurate predictions. Finally, the 3D reconstruction of tumors is facilitated through a spatial attention-based TLSTM network paired with a GAN, optimizing detail and fidelity in rendering tumor volumes.

Figure 2 shows the innovative framework of our suggested model, which includes three critical phases: lung segmentation, tumor detection, and 3D tumor reconstruction. The primary aim of lung segmentation is to achieve high performance in separating lung regions from surrounding thoracic structures within imaging data. This meticulous step is crucial as it ensures that the subsequent processes, tumor detection and 3D reconstruction, rely on accurate inputs. Our lung segmentation approach utilizes a GAN with U-Net architecture, specifically designed for detailed pixel-by-pixel categorization. However, this method encounters challenges due to class imbalance, which is the disproportionate representation of lung and non-lung pixels in the dataset, with non-lung pixels being more prevalent. To address this imbalance, we employ an off-policy PPO algorithm that prioritizes non-lung pixels over lung pixels. The off-policy characteristic of PPO enables the utilization of a wide spectrum of historical data, diminishing the dependency on potentially biased recent data.

Following lung segmentation, the next phase is tumor detection. This stage involves examining the segmented lung images to identify potential tumor regions. Here, we integrate a GAN alongside the Mask R-CNN framework. This powerful tool combines the capabilities of object detection and instance segmentation, which effectively identifies and localizes the affected areas within the segmented images for precise tumor detection and categorization. Additionally, an error correction mechanism is integrated into the loss function of the generator to identify and rectify any discrepancies between the generated images and actual CT scans, thus enhancing the quality of the detected regions.

The final stage of our proposed model is 3D tumor reconstruction. This process involves creating a 3D tumor model from the detected tumors. Our model for 3D tumor reconstruction employs a spatial attention-based TLSTM network, a type of recurrent neural network that can process data sequences while also considering the spatial relationships between the sequence elements paired with a GAN. The TLSTM network processes features extracted from 2D pulmonary scans by an EfficientNet network and uses transductive learning, adjusting its weights based on proximity to new data points, to significantly enhance the prediction of critical tumor-related features. The generator then creates a 3D model based on the outputs from the TLSTM network.

In all GANs, the role of the discriminator is to evaluate the images generated by the generator. Discriminator networks use dilated convolution layers, a convolutional layer that can expand the receptive field without compromising image resolution or adding significant computational overhead. This is especially valuable in processing high-resolution medical images, where maintaining detail is crucial. Dilated convolutions enable the discriminator to capture a wider context and finer details across larger image areas, more accurately differentiating between real and generated images.

### Lung segmentation

The primary goal of lung segmentation is to generate accurate lung contours that meticulously replicate the original reference outlines. In this paper, we employ a GAN for lung segmentation, which includes two networks: a generator and a discriminator. The generator excels at detecting essential features and patterns in the scans, creating outlines that accurately map the lung areas. These outlines are designed to capture the complex shapes and edges necessary for thorough segmentation. On the other hand, the discriminator works with the generator to enhance the quality of these outlines. Acting as a strict evaluator, the discriminator checks how well the generated outlines match the original ones. It uses the Earth Mover’s distance (EMD), a detailed measurement, to assess how much the generated outlines differ from the actual ones, helping to improve the outputs of the generator. This reciprocal evaluation process encourages the generator to create contours that better match the original, thus improving the segmentation quality. During training, the generator processes a lung CT scan image, denoted as $\:{I}_{i}$, and produces a corresponding outline $\:{M}_{i}$ that highlights the lung areas. The discriminator then examines this generated outline $\:{M}_{i}$ for its fidelity and quality, providing essential feedback that enhances the performance of the generator. This iterative cycle, where the generator and discriminator continuously interact and refer to the original outlines, is crucial in enhancing the quality of the lung contours produced during training.

#### Generator

**Fig. 3:**
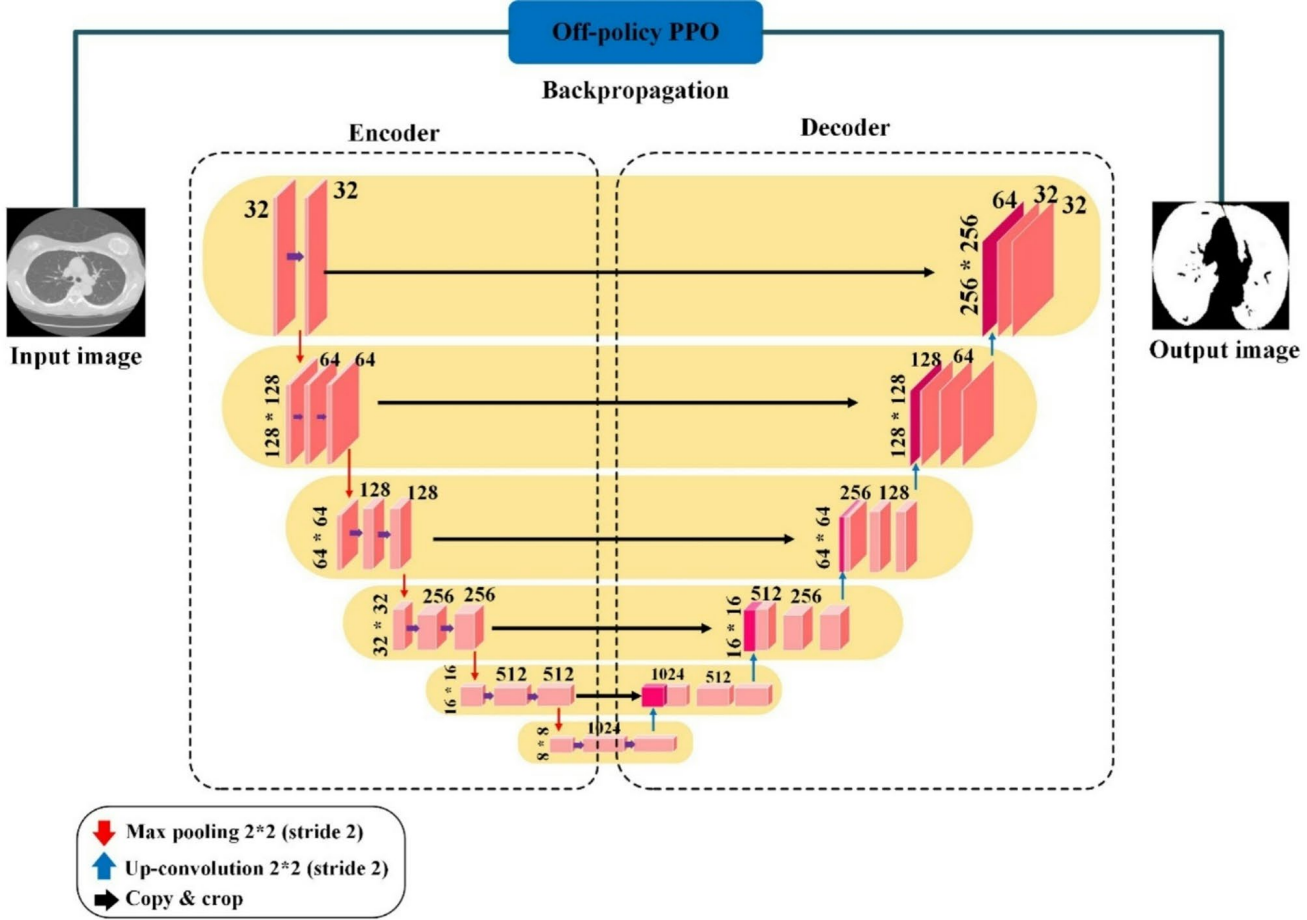
Architecture of U-Net-based generator for lung segmentation. This diagram illustrates the dual-component structure of the generator, consisting of an encoder and a decoder. The encoder processes the input CT scan image, extracting features at various scales through convolution layers to capture detailed information. The decoder then uses these features to construct precise masks delineating lung regions. In this model, the off-policy PPO algorithm is integrated, which enhances the ability of the model to classify each pixel accurately, addressing the challenge of class imbalance by prioritizing the detection of lung area pixels.

In our specific context, segmentation involves classifying each pixel in an image as belonging to the lung area. When processing a CT scan labeled $\:{I}_{i}$, the generator network assesses each pixel to determine whether it is part of the lung region, then creates a corresponding mask $\:{M}_{i}$ that reflects this classification. The structure of the generator is depicted in Fig. 3. The generator employs a U-Net for segmenting lungs, consisting of two main parts: the encoder and the decoder. The encoder, a key player in this process, receives the input image and extracts features at various scales via a sequence of convolution layers. These layers are intricately designed to grasp a wide spectrum of nuances and hierarchical details from the image with high fidelity. Following this, the decoder employs these hierarchical features to craft the masks, meticulously reconstructing the complex forms and contours of the lung regions to generate precise and detailed masks. The encoder and decoder incorporate convolution layers, which are essential in detecting local patterns and spatial correlations necessary for precise segmentation.

The generator acts as a binary pixel classifier, labeling each pixel as part of the lung area (marked as one) or not (marked as zero). The prevalence of non-lung pixels (zeroes) leads to a class imbalance, causing the classifier to favor predictions of non-lung areas and reducing its performance in identifying lung regions. To rectify this, in addition to adversarial loss, we implement an off-policy PPO algorithm in our training strategy, effectively addressing the imbalance. This algorithm, equipped with custom reward and penalty systems, optimally addresses this issue by offering higher rewards or penalties for correct or incorrect identifications of lung pixels (the minority class) compared to non-lung pixels (the majority class). This adjustment helps the model to concentrate more accurately on recognizing lung area pixels. By boosting incentives for correctly identifying the minority class, the off-policy PPO algorithm directs the focus of the generator toward lung areas, thus correcting the bias towards the abundant non-lung pixels. This strategic enhancement balances the focus of the classifier between both classes and significantly improves the performance of the model in segmenting lung regions from CT images.

In the proposed off-policy PPO algorithm, the framework for state, action, and reward is defined as follows:

- State $\:{s}_{t}$: It represents the observed pixel at a specific time step $\:t$.
- Action $\:{a}_{t}$: It is committed to forecasting the label of every pixel as lung or non-lung.
- Reward $\:{r}_{t}$: It reflects the effectiveness of a specific action. If an agent correctly classifies, it receives a positive incentive; if not, a negative one is issued. The magnitude of these incentives should vary between the classes. In this study, incentives for actions are determined based on the equation below:

18 $$\:{r}_{t}\left({s}_{t},{a}_{t},{y}_{t}\right)=\left\{\begin{array}{c}+1\:,{a}_{t}={y}_{t}\:and\:{a}_{t}\in\:{D}_{L}\\\:-1\:,{a}_{t}\ne\:{y}_{t}\:and\:{a}_{t}\in\:{D}_{L}\\\:+\lambda\:\:,{a}_{t}={y}_{t}\:and\:{a}_{t}\in\:{D}_{N}\\\:-\lambda\:\:,{a}_{t}\ne\:{y}_{t}\:and\:{a}_{t}\in\:{D}_{N}\end{array}\right.$$

Where $\:{D}_{L}$ and $\:{D}_{N}$​ represent the minority (lung) and majority (non-lung) classes, respectively. To tackle class imbalance, the reward function is asymmetrically designed to assign higher importance to correctly classifying minority (lung) pixels. Specifically, a correct classification from the minority class yields a strong positive reward (+ 1), while a misclassification incurs a strong penalty (–1). In contrast, the majority class receives lower-magnitude rewards or penalties ($\:\pm\:\lambda\:$), where $\:\lambda\:$ is a positive constant of less than one. This asymmetric weighting helps the model prioritize the minority class. It does so by assigning higher penalties for misclassifying lung pixels and greater rewards for correctly identifying them. This approach reduces the overwhelming influence of non-lung pixels during training.

**Fig. 4:**
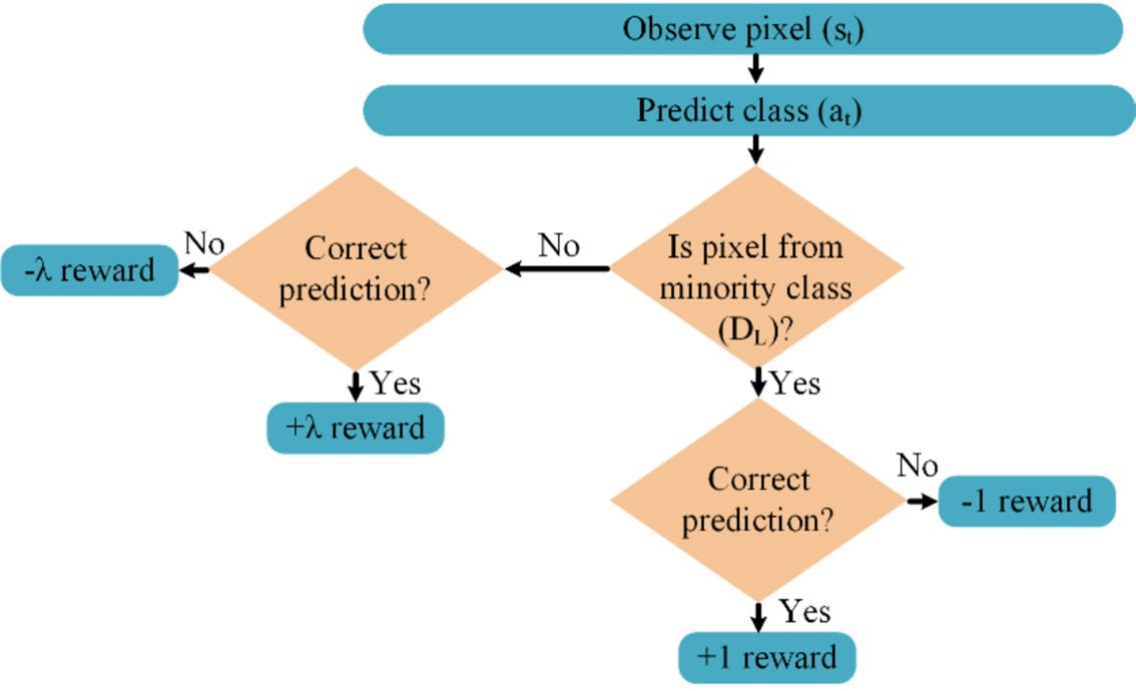
Flowchart illustrating the reward assignment strategy in the off-policy PPO algorithm for lung segmentation.

To better illustrate how the reward mechanism addresses class imbalance, consider the flowchart in Fig. 4. At each time step, the agent observes a pixel ($\:{s}_{t}$) and predicts a class label ($\:{a}_{t}$​). If the prediction is correct and the pixel belongs to the minority class ($\:{D}_{L}$​), a strong positive reward (+ 1) is issued. Conversely, if misclassified, a strong penalty (–1) is applied. For the majority class pixels ($\:{D}_{N}$​), correct predictions yield a small reward ($\:+\lambda\:$), while incorrect ones result in a small penalty ($\:-\lambda\:$). This reward design helps the model learn more accurate boundaries for lung pixels. It does this by assigning stronger penalties for mistakes in the minority class than in the majority class, as shown in the flowchart.

Algorithm 1 outlines the training process of the proposed generator for lung segmentation. This approach integrates both off-policy PPO and adversarial learning to address the severe class imbalance inherent in segmentation tasks. At each iteration, the generator is treated as a policy network that predicts whether each pixel belongs to the lung or non-lung class. A custom-designed reward function, detailed in Eq. 18, assigns asymmetric incentives to correctly or incorrectly classified pixels, prioritizing the underrepresented lung class. In parallel, the generator receives feedback from a discriminator network, which evaluates the anatomical plausibility of the generated segmentation masks. To incorporate both learning signals, the generator is updated using a composite loss function: the clipped PPO loss, which enhances stability in off-policy updates, and an adversarial loss that ensures structural realism in the outputs. These two loss components play complementary roles: while the PPO loss provides fine-grained, pixel-level guidance under class imbalance, the adversarial loss promotes structural plausibility at the global level. This synergy enables the generator to enhance both voxel-level precision and holistic structural realism, which is particularly crucial in medical segmentation tasks. Unlike traditional segmentation methods that rely solely on cross-entropy or Dice-based losses, our method introduces a reinforcement learning signal tailored to the minority class and couples it with a GAN-based discriminator. This novel combination yields a more robust segmentation of lung areas, particularly in challenging conditions with severe imbalances and anatomical variability.

**Algorithm 1:**
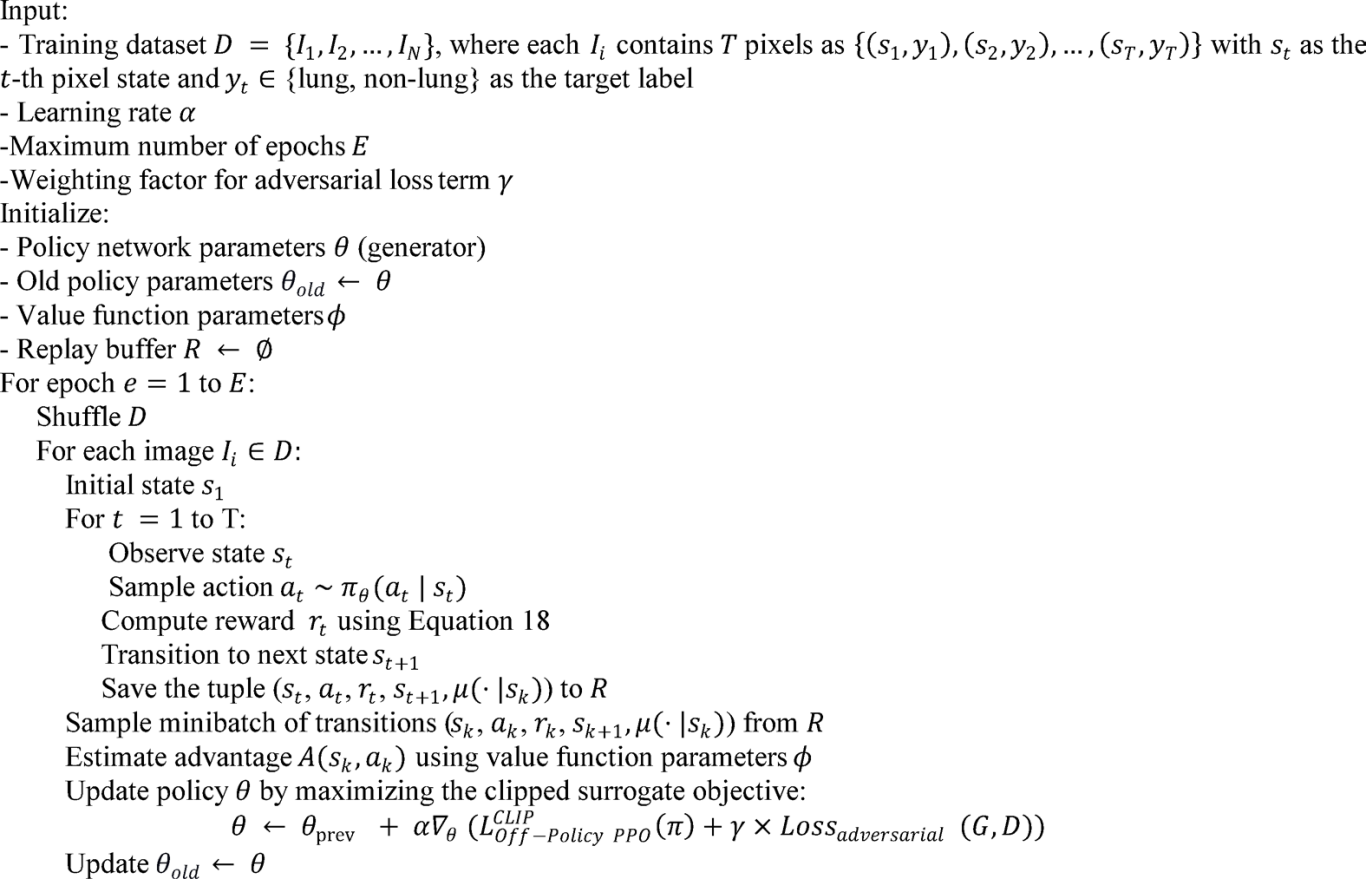
Pseudocode for training of the generator in lung segmentation.

##### Off-policy PPO

This research presents an advanced version of PPO known as off-policy PPO, which optimizes data utilization by integrating information from previous experiences and decisions. Diverging from the conventional on-policy PPO that depends solely on immediate environmental data for effective operation, off-policy PPO incorporates a broader, more diverse dataset. This dataset comprises both prior interactions and assorted tactical decisions. Consider, for instance, a robot navigating through a complex maze. In contrast, standard on-policy PPO leverages only current observations to refine its approach; off-policy PPO utilizes insights from prior navigational attempts and evaluates different strategies across varying scenarios. This method accelerates learning by minimizing the need for the robot to exhaustively explore each potential route, thereby improving efficiency by exploiting extensive historical data.

Off-policy PPO addresses the optimization challenge by enhancing a surrogate objective function through the utilization of off-policy data, mirroring the technique used in off-policy TRPO^97^:

19 $$\:\underset{\pi\:}{{max}}{E}_{s\sim{\rho\:}_{\mu\:},\:\:a\in\:\mu\:}\left[\frac{\pi\:\left(a|s\right)}{\mu\:\left(a|s\right)}{A}_{{\pi\:}_{old}}\left(s,a\right)\right]$$

subject to:

20 $$\:{\stackrel{-}{D}}_{KL}^{{\rho\:}_{\mu\:},sqrt}(\mu\:,{\pi\:}_{old}){\stackrel{-}{D}}_{KL}^{{\rho\:}_{\mu\:},sqrt}({\pi\:}_{old},\pi\:)+\:{\stackrel{-}{D}}_{KL}^{{\rho\:}_{\mu\:}}({\pi\:}_{old},\pi\:)\le\:\delta\:$$

where

21 $$\:{\rho\:}_{\mu\:}\left(s\right)=\sum\:_{t=0}^{\infty\:}{\gamma\:}^{t}P({s}_{t}=s|{s}_{0},\mu\:)$$

22 $$\:{\stackrel{-}{D}}_{KL}^{{\rho\:}_{\mu\:}}\left({\pi\:}_{old},\pi\:\right)={E}_{s\sim{\rho\:}_{\mu\:}}\left[{D}_{KL}\left({\pi\:}_{old}\left(.|s\right)\:\left|\right|\:\pi\:\left(.|s\right)\right)\right]$$

23 $$\:{\stackrel{-}{D}}_{KL}^{{\rho\:}_{\mu\:},sqrt}\left(\mu\:,{\pi\:}_{old}\right)={E}_{s\sim{\rho\:}_{\mu\:}}\left[\sqrt{{D}_{KL}\left(\mu\:\left(.|s\right)\:\left|\right|\:{\pi\:}_{old}\left(.|s\right)\right)}\right]$$

24 $$\:{\stackrel{-}{D}}_{KL}^{{\rho\:}_{\mu\:},sqrt}\left({\pi\:}_{old},\pi\:\right)={E}_{s\sim{\rho\:}_{\mu\:}}\left[\sqrt{{D}_{KL}\left(\:{\pi\:}_{old}\left(.|s\right)\:\left|\right|\:\pi\:\left(.|s\right)\right)}\right]$$

Here, $\:\mu\:$ denotes the behavior policy. Absent the constraints imposed by Eq. 20, striving to enhance the surrogate function using off-policy data (outlined in Eq. 19) could result in extensive policy modifications. To curtail this hazard, the PPO clipping mechanism is utilized to aptly refine the surrogate objective^97^:

25 $$\:{L}_{\mu\:}\left(\pi\:\right)={E}_{s\sim{\rho\:}_{\mu\:},\:\:a\in\:\mu\:}\left[\frac{\pi\:\left(a|s\right)}{\mu\:\left(a|s\right)}{A}_{{\pi\:}_{old}}\left(s,a\right)\right]$$

Using $\:{L}_{\mu\:}\left(\pi\:\right)$, we establish the clipped surrogate objective for off-policy data as delineated below^97^:

26 $$\:\overline{L} _{\mu } \left( \pi \right) = E_{{s\sim \rho \:_{{\mu {\kern 1pt} }} ,\:\:a \in \:\mu \:}} \left[ {{\text{min}}\left( {\frac{{\pi \:\left( {a|s} \right)}}{{\mu \:\left( {a|s} \right)}}A_{{\pi \:_{{old}} }} \left( {s,a} \right),clip\left( {\frac{{\pi \:\left( {a|s} \right)}}{{\mu \:\left( {a|s} \right)}},1 - \in \:,1 + \in \:} \right)A_{{\pi \:_{{old}} }} \left( {s,a} \right)} \right)} \right]$$

The proportion $\:\frac{\pi\:\left(a|s\right)}{\mu\:\left(a|s\right)}$ often surpasses the bounds of $\:1\:-\:\epsilon\:$ and $\:1\:+\:\epsilon\:$, which typically leads to the policy $\:\pi\:\left(a|s\right)$ remaining unchanged while optimizing the clipped surrogate objective. To counter this stagnation, the limits of the clipped objective, $\:\left(\right(1\:-\:\epsilon\:),\:(1\:+\:\epsilon\:\left)\right)$, are modified in Eq. 26 by incorporating a correction factor $\:\frac{{\pi\:}_{{\theta\:}_{i}}\left(a|s\right)}{\mu\:\left(a|s\right)}$^97^:

27 $$\begin{aligned} L_{{Off{\text{-}}Policy~PPO}}^{{CLIP}} \left( \pi \right) & = E_{{s\sim \rho _{\mu } ,~~a \in \mu }} \left[ {\min \left[ {\frac{{\pi \left( {a{\text{|}}s} \right)}}{{\mu \left( {a{\text{|}}s} \right)}}A_{{\pi _{{old}} }} \left( {s,a} \right),} \right.} \right. \\ & \quad \left. {\left. {clip\left( {\frac{{\pi \left( {a{\text{|}}s} \right)}}{{\mu \left( {a{\text{|}}s} \right)}},\frac{{\pi _{{old}} \left( {a{\text{|}}s} \right)}}{{\mu \left( {a{\text{|}}s} \right)}}\left( {1 - \in } \right),\frac{{\pi _{{old}} \left( {a{\text{|}}s} \right)}}{{\mu \left( {a{\text{|}}s} \right)}}\left( {1 + \in } \right)} \right)A_{{\pi _{{old}} }} \left( {s,a} \right)} \right]} \right] \\ \end{aligned}$$

**Fig. 5:**
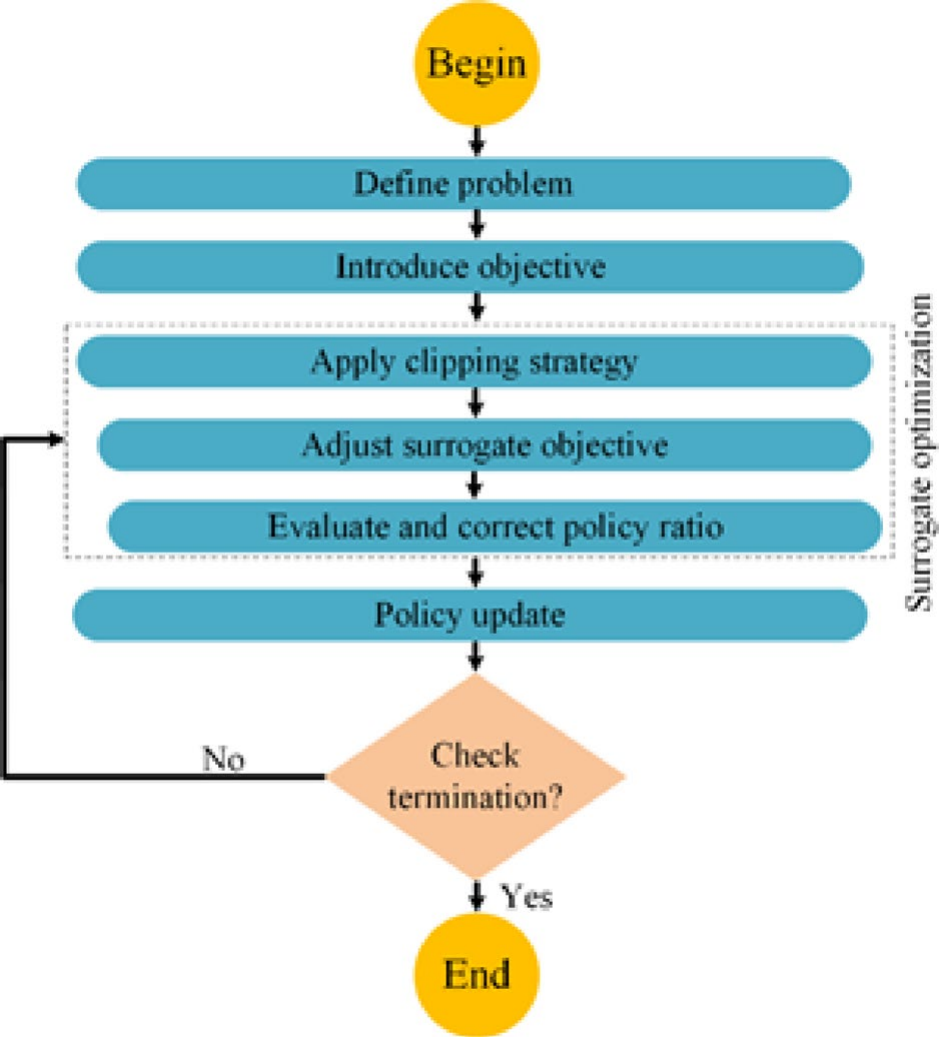
Policy optimization for lung segmentation using off-policy PPO in the U-Net framework.

Figure 5 presents a streamlined seven-stage framework for optimizing the lung segmentation model using the off-policy PPO algorithm. The process begins by defining the optimization objective within a U-Net environment. A simplified surrogate objective is used along with a clipping mechanism to prevent drastic changes in the policy. The objective is then modified to incorporate previously collected data, enabling the model to generalize more effectively. The clipped policy ratio is evaluated and corrected if it exceeds the allowed range, ensuring stable and gradual learning. The final policy is updated iteratively until convergence is reached. Key steps are highlighted to clarify logical progression and reduce visual complexity.

#### Discriminator

The discriminator in the lung segmentation stage is designed to distinguish between real segmented masks and those generated by the GAN-based segmentation network. It receives binary segmentation masks as input. Then, it applies several dilated convolutional layers to extract multi-scale contextual features. These features are crucial for determining whether the segmentation is anatomically accurate. Instead of comparing pixels directly, the architecture allows the discriminator to evaluate whether the entire structure and shape are consistent with real anatomical forms. The output is a spatial probability map that indicates the realism of each region in the input mask. This map helps localize inconsistencies in shape or structure that the generator needs to correct. During training, the EMD is used as an additional evaluation metric. EMD measures the difference between the real and synthetic masks across spatial regions. This goes beyond simple pixel-level errors. It encourages the generator to learn the spatial arrangement and anatomical shape of the lungs more precisely. Together, these mechanisms refine the generator output toward more realistic, anatomically aligned segmentation masks.

In duties such as image synthesis with GANs, the objective is usually to produce images that are indistinguishable from genuine ones. Nevertheless, lung segmentation offers distinct challenges. Authentic masks are binary, consisting only of 0s and 1s, but the masks produced by the generator vary continuously from 0 to 1. This difference may lead the discriminator to overly focus on identifying real versus produced masks, overlooking other vital factors.

To address the challenge, we have adopted a sophisticated approach. Instead of directly feeding the fake or genuine masks within the discriminator, we incorporate lung slice images into the input. These images are modified versions of the CT scans, adjusted with segmented and real masks. This method enables the discriminator to assess the connection between the produced masks and the lung images, thereby enhancing its capacity to accurately differentiate between genuine and produced masks. Further, a specialized segmentation mask that selectively emphasizes nodule areas while setting other areas to zero is used. This extra detail helps the discriminator better recognize essential features, improving its differentiation capability. The loss function for the discriminator is defined in the following equation:

28 $$\:{E}_{x\sim\:{p}_{z}}\left[D\right(G\left(x\right)\:\text{o}\:{I}_{i}\left)\right]-{E}_{x}\sim\:{p}_{real}\left[D\right(x\left)\right]$$

In this context, $\:{p}_{z}$ and $\:{p}_{real}$ show represent the distributions of the synthetic and real data, respectively. The symbol $\:o$ denotes an operator that carries out pixel-wise multiplication. This operation merges the intensity data from the CT images with the spatial data from the masks. It compels the discriminator to evaluate the consistency of the fused information instead of assessing the components separately.

### Tumor detection

**Fig. 6:**
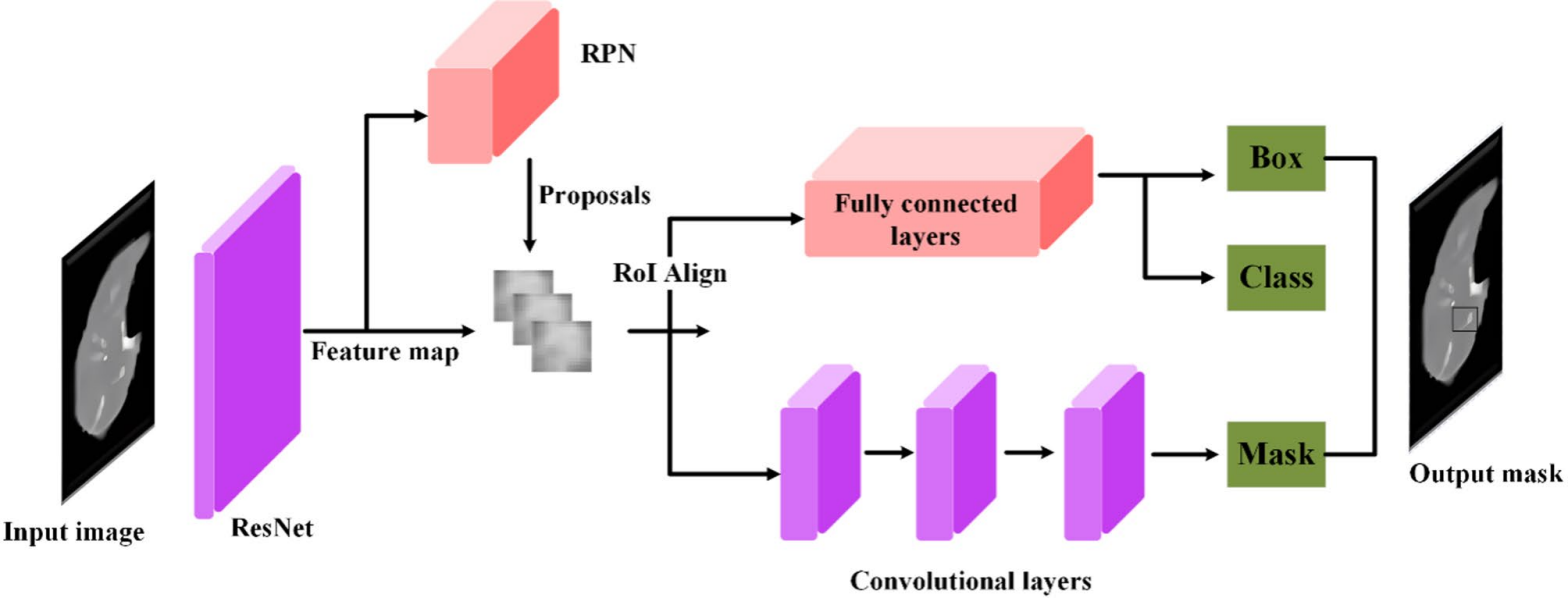
Architecture of the Mask R-CNN-based generator network for tumor detection.

Figure 6 illustrates the structure of the Mask R-CNN-based generator network for tumor detection. In this paper, our model adopts a more targeted strategy than the traditional GAN approach of generating synthetic outputs from random noise. It employs lung data from a previous step as input, sidestepping the randomness typical of conventional GANs. This deliberate input selection empowers the generator to fully exploit the comprehensive details in the lung data, producing accurate and appropriate outputs. The primary aim of the generator is to generate outputs that offer classification and precise location information. During the analysis of lung imagery, the generator categorizes various areas within the lungs, accurately labels them, and calculates their boundaries using the coordinates of the bounding box.

We employ a novel loss function, as described in Eq. 29, to guide the training of the generator. This function combines classification loss, bounding box regression loss, and an adversarial term.

29 $$\:{L}_{G}={L}_{cls}+\:{L}_{box}+{L}_{adv}^{{G}_{b}}$$

where $\:{L}_{cls}$ and $\:{L}_{box}$ represent the standard classification and bounding box regression losses as used in the Mask R-CNN method. These ensure accurate class labeling and box placement. However, many existing models rely solely on these two losses, limiting their ability to capture realistic tumor structures in ambiguous or irregular regions. The adversarial loss $\:{L}_{adv}^{{G}_{b}}$ in Eq. 30 addresses this gap:

30 $$\:{L}_{adv}^{{G}_{b}}=\frac{1}{N}\sum\:_{i=1}^{N}-{log}{D}_{b}\left({G}_{b}\left({RoI}_{i}\right)\right)\:\:$$

where $\:N$ indicates the mini-batch size, and $\:{G}_{b}\left({RoI}_{i}\right)$ refers to the prediction of the $\:i$-th bounding box. This adversarial component drives the model to generate bounding boxes that reflect the high-level semantic structure of tumor regions, leading to more robust predictions in cases with low contrast or morphological ambiguity. The integration of classification and regression losses ensures core detection accuracy, while the adversarial term refines structural plausibility. Together, these components guide the model to locate tumors and outline their shapes more accurately, even when the intensity information is unclear or varies across regions.

The loss function of the discriminator, detailed in Eq. 31, employs CNNs to enhance its capacity to distinguish between genuine and artificial bounding boxes, thus improving the effectiveness of the training process.

31 $$\:{L}_{D}=\frac{1}{N}\sum\:_{i=1}^{N}-[{log}({D}_{b}\left({bb}_{i}^{{g}_{t}}\right))+\:log(1-{D}_{b}\left({G}_{b}\left({RoI}_{i}\right)\right)\left)\right]$$

where $\:{D}_{b}$ reflects the probability that the image is genuine. The adversarial loss $\:{L}_{adv}^{{G}_{b}}$ motivates $\:{G}_{b}$ to generate bounding boxes convincing enough to deceive $\:{D}_{b}$. This loss term is designed to encourage the generator to produce increasingly convincing boxes, making them indistinguishable from real ones and thus narrowing the gap between synthetic and authentic distributions.

**Fig. 7:**
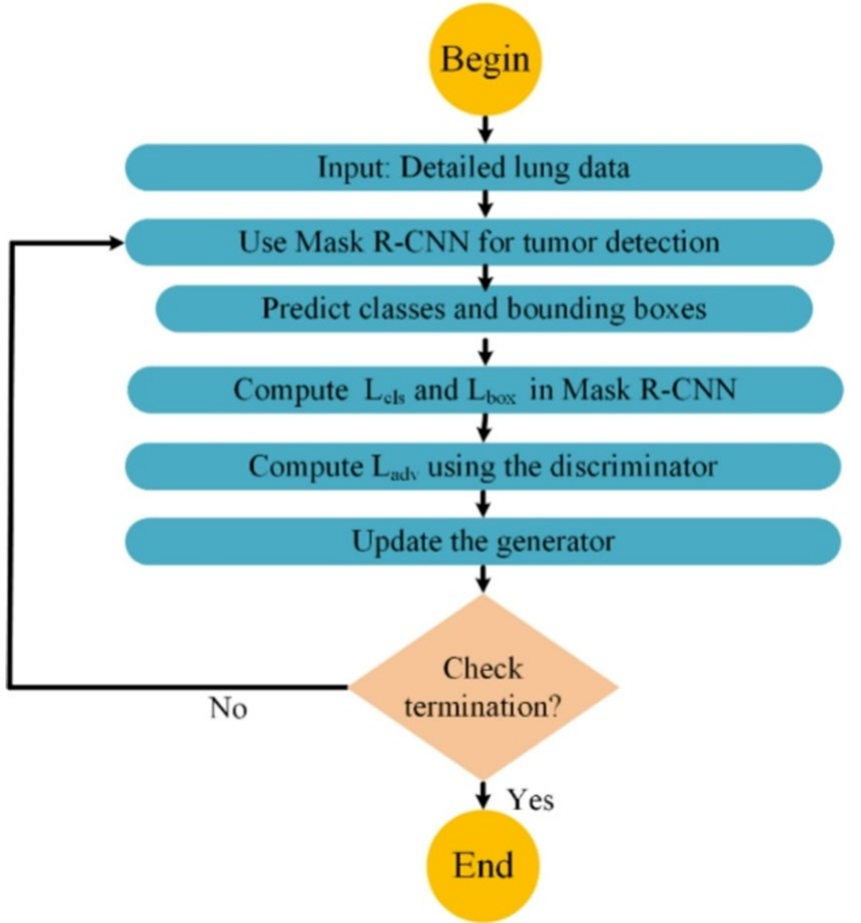
Generator training for tumor detection using Mask R-CNN with adversarial loss.

Figure 7 depicts the optimization flowchart for the generator used in tumor detection within the Mask R-CNN framework. The process starts by inputting segmented lung data essential for training the generator to accurately recognize and classify features in lung images. The Mask R-CNN then processed this data. In this system, the generator identifies the categories of different regions (either tumor or non-tumor) and computes bounding box coordinates that accurately pinpoint tumors in the lung scans. During training, two critical loss components are calculated: the class loss ($\:{L}_{cls}$) evaluates the performance of tumor classifications, and the bounding box loss ($\:{L}_{box}$) assesses the quality of the bounding boxes surrounding the detected tumors. These metrics provide essential feedback for refining the performance of the generator. Additionally, the discriminator calculates the adversarial loss ($\:{L}_{adv}$) to determine how closely the synthetic images mimic actual lung scans. This step is vital as it drives the generator to produce images that resemble real medical scans, thus enhancing the clinical applicability of the model. The optimization includes a feedback loop, continuously updating the generator based on these losses to enhance quality and realism. This prediction, loss evaluation, and updates cycle persists until the model achieves predefined termination criteria, such as reaching a maximum number of iterations. This methodical process ensures that each step boosts the efficacy of the generator, resulting in a powerful model adept at detecting tumors in lung CT images.

The discriminator in the tumor detection stage is designed to evaluate the quality of bounding boxes and classifications produced by the generator. It takes as input the feature maps of predicted tumor regions, especially the RoIs) generated by the Mask R-CNN head. Instead of relying only on coordinate accuracy, the discriminator checks whether the predicted regions appear realistic based on semantic and anatomical information. It applies dilated convolutional layers to capture contextual features at different scales. This helps the discriminator assess the overall structure and coherence of the predicted tumor regions. The output is a scalar probability that reflects how closely a predicted region matches real tumors. During training, the EMD is employed as an additional loss function to quantify the differences between real and generated tumor distributions. In contrast to the segmentation stage, where the discriminator evaluates entire binary masks, this stage operates at the RoI level. It compares the semantic features of predicted bounding boxes with those of real tumor annotations. This setup enables the generator to improve both the accuracy of tumor localization and the realism of the predicted structures. The combined use of EMD and dilated convolutions ensures that feedback from the discriminator guides the generator toward more anatomically faithful tumor detection.

### 3D reconstruction method

As shown in Fig. 3, the 3D reconstruction stage processes $\:N$ sequences of output images from the tumor detection stage. These images undergo feature extraction through the pre-trained EfficientNet network, which is known for its effectiveness. The architecture of EfficientNet refined through learning from the vast data, ensures high performance in recognizing and categorizing image content. Its layers capture a wide range of features, from simple edges in the early layers to complex object parts in deeper layers, making it highly suitable for medical imaging where fidelity is critical. The extracted features are then fed into TLSTM units, crucial for enhancing temporal continuity in the data. By integrating context from previous frames, TLSTM units greatly enhance the interpretation of the current frame.

A spatial attention mechanism is placed before the TLSTM units to improve their ability to capture key temporal features. This mechanism enables the model to focus selectively on the most informative parts of each input image rather than treating all spatial regions equally. The attention layer is trained to prioritize features that are essential for 3D reconstruction. These often include tumor boundaries or prominent anatomical structures. By emphasizing these critical areas, the model reduces the influence of background noise and less significant regions, which often distract conventional sequence models. This focused representation enables TLSTM to interpret each frame within the context of both previous frames and spatial relevance, resulting in more coherent and anatomically accurate 3D reconstructions.

**Fig. 8:**
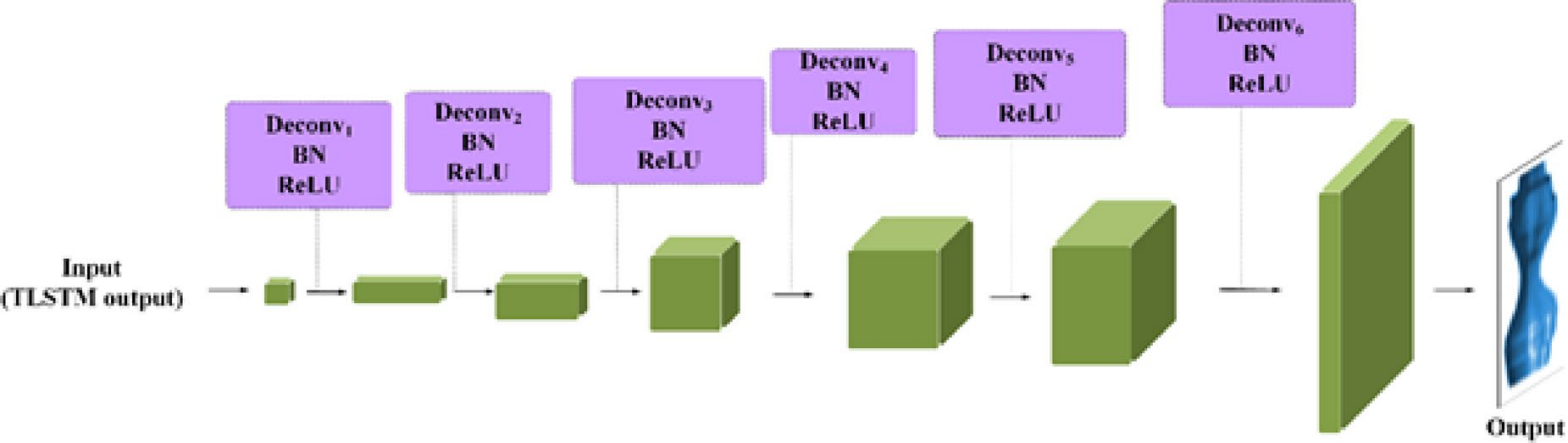
Architecture of the generator network for the 3D reconstruction of lung tumors.

Figure 8 illustrates the structure of the generator network, which directly receives the output from the TLSTM module. The TLSTM extracts features that remain consistent over time from the image sequence and incorporates spatial attention to emphasize important regions in each frame. These features are reshaped into a compact 3D tensor that retains anatomical continuity across slices. This 3D tensor serves as the starting input to the generator, forming the basis for volumetric reconstruction. The generator processes this input, which contains both temporal and spatial information, to produce outputs with greater accuracy and more consistent anatomical structure.

The architecture of the generator features six deconvolution layers, each equipped with batch normalization (BN) and the ReLU activation function. In the GAN setup, the discriminator plays a crucial role. It accurately differentiates synthetic and real-world images from the generator, ensuring the 3D images are realistic. This effectiveness is vital for correct medical diagnosis and planning treatments. By combining the powerful feature extraction of EfficientNet with advanced sequence processing and generative modeling, this method successfully creates precise 3D representations from 2D image sequences, greatly improving the quality and realism of the generated images.

The discriminator in the 3D reconstruction stage is designed to evaluate the realism of volumetric images generated by the GAN-based architecture. It receives as input the reconstructed 3D lung tumor volumes produced by the generator. The discriminator compares these generated volumes with the corresponding real, annotated 3D ground-truth data to evaluate the structural authenticity. To evaluate these 3D volumes, the discriminator employs 3D dilated convolutional layers. This structure enables it to analyze spatial dependencies in all three dimensions and detect inconsistencies in the generated structures. The output is a scalar prediction score that reflects the anatomical plausibility of the reconstructed volume. During training, EMD is employed as a global evaluation criterion, quantifying the distributional shift between real and synthetic 3D data across spatial regions. In contrast to earlier stages, where the discriminator evaluates 2D binary masks or region proposals, it operates on entire 3D reconstructions here. The discriminator acts not only as a classifier but also as a spatial coherence assessor, ensuring the generated volumes exhibit consistent shape, texture, and continuity over slices. This feedback loop encourages the generator to refine outputs that are both anatomically consistent and visually coherent in the volumetric space.

Algorithm 2 shows the training procedure for the proposed TLSTM-based 3D tumor reconstruction model. The process begins with a training dataset composed of sequences of 2D tumor-detected slices, each annotated with a corresponding 3D ground-truth volume. Each slice from the input sequence is processed by a pre-trained EfficientNet encoder. This step extracts important high-level features from each image. A spatial attention mechanism is then applied to enhance the focus on anatomically relevant regions such as tumor boundaries. These focused features are sent to a TLSTM module. This module updates its hidden and cell states at each step to preserve temporal consistency across the slices.

**Algorithm 2:**
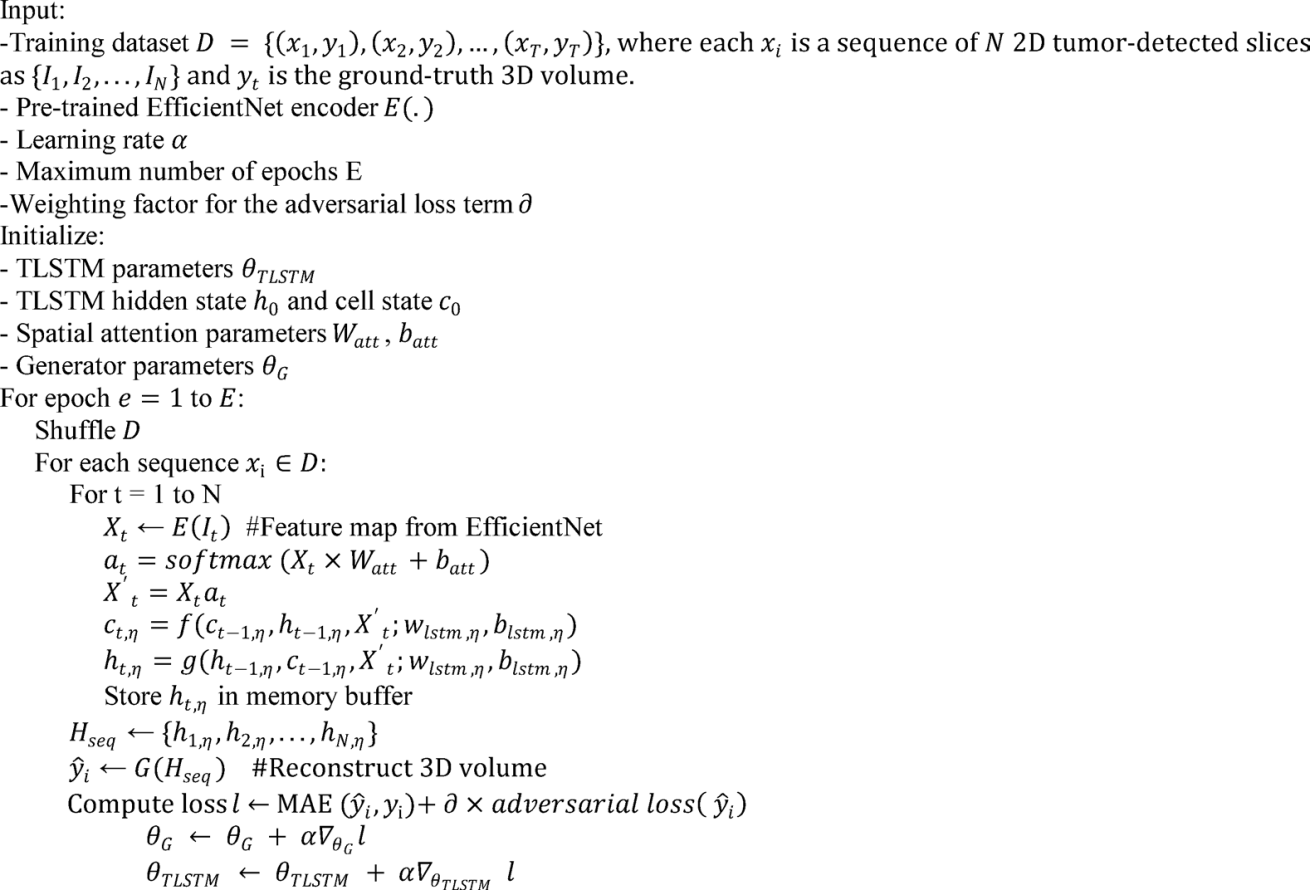
Pseudocode for training of TLSTM-based 3D tumor reconstruction.

The hidden state sequence contains both spatial and temporal information. This sequence is passed to a generator network that reconstructs the full 3D tumor volume. The training loss comprises two components: the mean absolute error (MAE) between the predicted and actual volumes and an adversarial loss. The adversarial loss is scaled using a hyperparameter. Gradients are backpropagated to update the parameters of the generator and the TLSTM units. This process gradually improves the quality of 3D reconstructions throughout training epochs.

### Overall algorithm

**Fig. 9:**
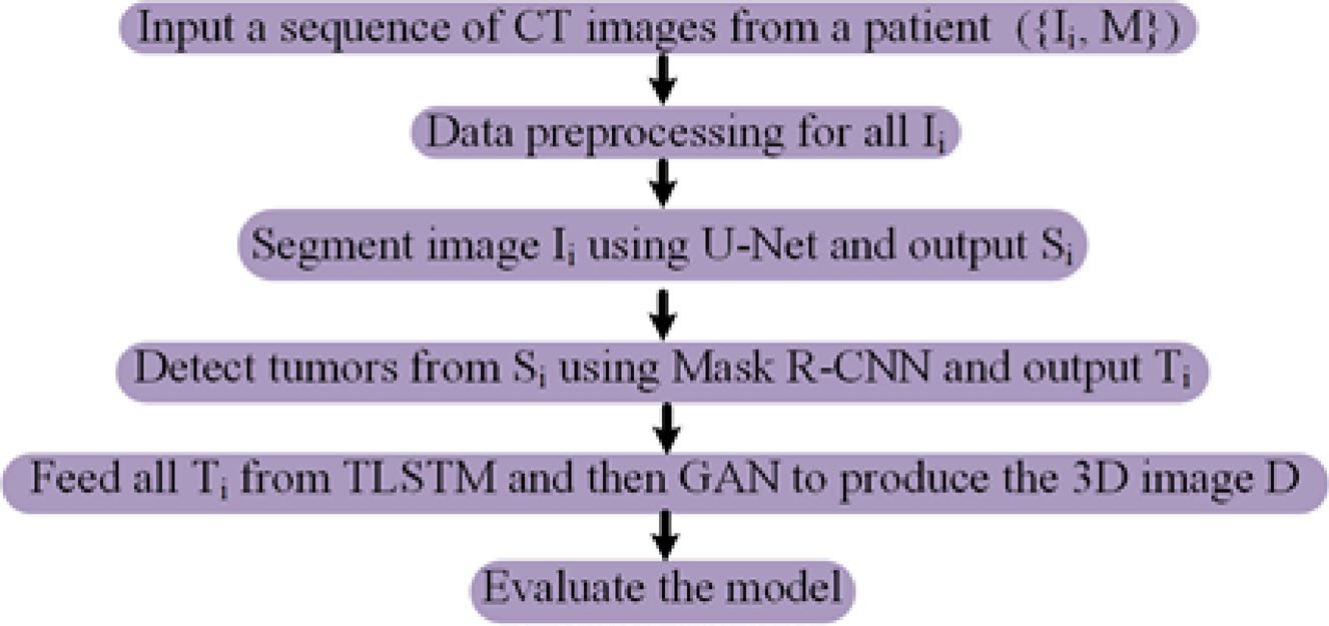
Workflow for the 3D reconstruction of lung tumors.

Figure 9 provides a comprehensive overview of the 3D reconstruction of lung tumors, outlining the step-by-step process from initial CT image acquisition to the final evaluation of the reconstructed 3D model. The flowchart starts with the input of a series of CT images from a patient, labeled as $\:\{{I}_{i},\:M\}$, where $\:M$ is the target 3D image derived from the sequence of $\:{I}_{i}$ images. This target image is crucial as it serves as the benchmark for assessing the performance of the reconstructed 3D model. The rest of the stages are shown in the following:

- Preprocessing stage: Initially, all CT images $\:{I}_{i}$ undergo extensive preprocessing to standardize the data and enhance image quality for more effective subsequent analysis. This includes resizing images to ensure consistent resolution across all inputs, which helps to standardize the data for better processing in later stages. Further preprocessing include normalization to adjust intensity scales and augmentation to increase the dataset variably and robustly by creating modified versions of existing images. These preprocessing efforts are vital for optimizing the performance of later stages, such as segmentation and detection.
- Segmentation process: Following preprocessing, each image $\:{I}_{i}$ is segmented using U-Net architecture. This neural network is particularly effective for medical image segmentation, producing an output $\:{S}_{i}$ delineating lung regions. These segmented images isolate the lung fields, enhancing clarity and focus for tumor detection.
- Tumor detection: The segmented images $\:{S}_{i}$ then undergo further analysis using Mask R-CNN, an algorithm tailored for precise object detection and instance segmentation. This step accurately identifies and localizes tumors within the lung segments, generating $\:{T}_{i}$ outputs that include detailed bounding boxes around each detected tumor.
- 3D reconstruction: After detecting the tumors, the relevant data $\:{T}_{i}$ is processed through a TLSTM and GAN system. The TLSTM network manages temporal relationships within the sequence data, refining the feature extraction, which is crucial for the subsequent synthesis by the GAN. The GAN then compiles these features to form a cohesive 3D image $\:D$, representing a detailed reconstruction of the lung tumors based on the 2D inputs.
- Model evaluation: The final evaluation stage compares the generated 3D image $\:D$ against the target 3D image $\:M$ to verify the performance of the model. This comparison is essential for confirming the clinical utility of the model by assessing the fidelity of the tumor structures in the 3D reconstruction. Ensuring the performance of these representations is critical for their potential application in diagnostic and treatment planning contexts.

## Empirical evaluation

In the following section, we will outline the dataset, describing its features and the scope of our study. This explanation will be followed by a discussion on the metrics used, where we will define the criteria and measurements employed to evaluate the performance of our models. The section will conclude with the presentation of results, spotlighting the primary outcomes from our analysis and model evaluations. We will also discuss the importance of these findings in our research objectives, offering insights into their implications and relevance to the field.

### Dataset

This article uses the LIDC-IDRI dataset^2^ to evaluate the proposed model, chosen for its relevance and applicability in real-world clinical scenarios. The LIDC-IDRI dataset is widely recognized in the medical imaging community for its comprehensive collection of annotated thoracic CT scans, which include expert-reviewed radiological annotations. This makes it an ideal choice for validating advanced imaging models like ours. Employing this well-established dataset guarantees robust evaluation metrics and benchmarks the performance of the model against a realistic standard. Furthermore, by matching the model with real-world clinical data and challenges, the dataset aids in evaluating the practical effectiveness and adaptability of the model to actual medical settings. This alignment helps demonstrate the potential of the model for deployment in healthcare systems, enhancing its credibility and utility in clinical settings.

The LIDC-IDRI, meticulously developed by the Foundation for the National Institutes of Health (FNIH) and the Food and Drug Administration (FDA), comprises 1,018 CT scans from 1,010 registered patients, accompanied by an extensible markup language (XML) file detailing annotations by four radiologists. The primary aim of this rigorous annotation process, conducted in two stages, is to accurately identify and document all nodules within each CT scan. The initial stage, blinded-read, involves every radiologist reviewing the scans to identify lesions. These are categorized as either “nodule < 3 mm” or “non-nodule ≥ 3 mm”. In the unblinded-read stage, radiologists revisit their annotations with access to the anonymous markings of their colleagues, fostering a collaborative environment for cross-verification and adjustments if necessary. Within this dataset, 7,371 lesions have been identified as nodules by at least one radiologist. Of these, 2,669 nodules are labeled consistently as ‘nodule ≥ 3 mm’ by all four radiologists, with 928 receiving unanimous agreement. These 2,669 nodules are further detailed with ratings based on their characteristics, such as size, shape, and density, and outlined visually in the dataset, enhancing their utility for diagnostic and research purposes.

It should be noted that the LIDC-IDRI dataset originally contained annotated 2D CT slices rather than native 3D volumes. To overcome this limitation, we developed a reproducible pipeline to generate consistent 3D volumes. This pipeline was implemented using Rhinoceros 3D (version 7)^98^, a widely adopted tool for 3D modeling and visualization. First, each annotated sequence of 2D slices corresponding to a single nodule case was imported into Rhinoceros using the Grasshopper plugin. A custom Python script was used to automatically extract nodule contours from each slice. These contours were then converted into closed polylines. The contours were stacked along the Z-axis based on the original CT slice thickness (typically 1.25 mm). A lofting operation was then applied to interpolate surfaces between the stacked contours. This process produced a smooth 3D mesh of the lesion. To enhance anatomical realism, the mesh was smoothed using the “Smooth Mesh” function with a setting level of 2 while preserving control points. The mesh resolution was set to medium, and a closed solid was generated for accurate volume rendering. Finally, all models were exported in standard .STL and .OBJ formats for downstream analysis. These settings ensure consistency and reproducibility in the 3D reconstruction process. The reconstructed models enrich the dataset by adding realistic 3D structures. They also serve as reliable ground truth for evaluating the performance of the proposed TLSTM-based volumetric reconstruction method.

To ensure consistency and improve model performance, several preprocessing steps were applied to the raw CT images before feeding them into the segmentation, detection, and reconstruction pipelines. First, slice alignment was applied to preserve spatial coherence across the image sequences. Slices were aligned along the same anatomical axis using DICOM metadata to prevent positional drift. Next, z-score normalization was used to standardize the intensity values in each image. This process ensured zero mean and unit variance, helping to reduce variability caused by different scanners or individual patient characteristics. To increase robustness and prevent overfitting, various data augmentation methods were applied during training. These included random rotations (± 15°), horizontal and vertical flipping, and elastic deformations to simulate realistic anatomical variations. All preprocessing steps were implemented in Python using the SimpleITK and Albumentations libraries. These operations were applied uniformly across the training and validation subsets, significantly contributing to the reproducibility and generalizability of the model across different data distributions.

We separated the data for all models into training, validation, and testing subsets to streamline model training and evaluation. For lung segmentation and tumor detection using the LIDC-IDRI dataset, which includes 244,527 images, we distributed 70% of these images to the training subset, 15% to the validation subset, and 15% to the test subset. Specifically, this allocation provided approximately 171,169 images for training, 36,679 for validation, and 36,679 for testing. In the case of the 3D reconstruction model, this 70-15-15 distribution resulted in 5,785 images for training, 1,240 for validation, and 1,240 for testing. We chose this particular 70-15-15 split to ensure a balance between having ample data for training to enhance model learning and sufficient data for validation and testing to confirm thorough evaluation. By allocating 70% of the data to training, we ensure that the model learns from diverse examples, which helps it detect complex patterns and improve its generalization. The 15% of data set aside for validation is sufficient to adjust hyperparameters and prevent overfitting, enhancing the performance of the model on new data. Finally, the 15% allocated to testing offers an unbiased evaluation of the performance of the model, providing reliable insights into its effectiveness in real-world applications.

### Metrics

In evaluating lung segmentation and tumor detection models, intersection over union (IoU) and HD are used because these metrics help gauge the quality of the model and the boundaries it predicts. IoU is particularly critical for lung segmentation and tumor detection as it provides a clear measure of overlap consistency between the predictions of the model and the ground truth data. By calculating the intersection ratio to the union of the predicted and actual segments, IoU directly indicates how well the model can identify relevant areas within the lungs and tumors, regardless of their size or shape. This makes it invaluable in clinical settings where even small inaccuracies in segmentation can lead to significant differences in diagnosis or treatment outcomes. On the other hand, HD is essential for assessing the maximum distance between the predicted and true boundaries. It is vital as it confirms the exactness of the method in outlining the outer edges of the important regions. HD is beneficial when high exactness is required in capturing the boundaries of lung tissues and tumors, as slight deviations in these boundaries can impact clinical decisions like surgical margins or radiation targeting. Together, IoU and HD provide a comprehensive evaluation framework. They not only measure how much of the target the model successfully captures (IoU) but also how accurately it traces the most extreme perimeters of the targets (HD). This dual assessment helps ensure that the models are robust and effective for practical application in medical diagnostics and treatment planning, where overall robustness is critical.

In evaluating the 3D reconstruction model, HD and ED are chosen for their ability to effectively measure spatial fit and alignment errors. HD is particularly valuable as it measures the largest distance between the nearest points on the reconstructed surface and the actual model. It provides a worst-case scenario essential for high-performance applications like medical imaging and surgical planning. ED offers a simple geometric measure of the average distance between corresponding points on the actual and reconstructed models, helping assess the overall error throughout the volume. This combination ensures a robust evaluation, capturing both outlier errors and overall fidelity, which is essential for verifying the clinical reliability of the reconstructed 3D models. These metrics, together, provide a comprehensive view of the performance of the model in replicating intricate anatomical structures, which is vital for ensuring the realism required in medical applications.

The definitions for the metrics IoU, HD, and ED are provided as such:

- IoU is measured by the intersection ratio to the union of the pixel sets $\:A$ and $\:B$, where $\:A$ represents the pixels within the actual mask, and $\:B$ denotes the pixels within the predicted mask. These sets consist of non-zero pixels indicating the segmented areas.
  32 $$\:IoU=\frac{|A\cap\:B|}{|A\cup\:B|}$$
- HD quantifies the maximum distance from any point within one set to the nearest point in the alternative set. In this metric, $\:\left\| {a - b} \right\|$ represents the ED between points $\:a$ and $\:b$, which are points from sets $\:A$ and $\:B$, respectively.
  33 $$\:HD\:(A,\:B)=\text{max}\:(h\left(A,B\right),\:h\:(B,\:A\left)\right)$$
  with
  34 $$\:h\left( {A,B} \right) = \:\mathop {{\text{max}}}\limits_{{a\: \in \:\:A}} \mathop {{\text{min}}}\limits_{{b\: \in \:\:B}} \left\| {a - b} \right\|$$
- ED measures the straight-line distance between corresponding points in 3D space, denoted as $\:({x}_{1},{y}_{1},{z}_{1})$ and $\:({x}_{2},{y}_{2},{z}_{2})$. This metric provides a scalar value that reflects the positional alignment of each corresponding point pair in the reconstructed 3D models.
  35 $$\:ED=\sqrt{{({x}_{2}-{x}_{1})}^{2}+{({y}_{2}-{y}_{1})}^{2}+{({z}_{2}-{z}_{1})}^{2}}$$

**Table 5:** Optimal hyperparameter settings for the proposed method using cross-validation.

Hyperparameter description	Valid ranges	Best value
Long segmentation		
Rate of learning for generator	0.00001–0.01	0.002
Layers in generator	3–10	6
Rate of learning for discriminator	0.00001–0.01	0.001
Layers in discriminator	3–10	8
Size of batch	8–512	68
Momentum	0.6–0.9	0.85
Decay of weights	0. 001–0.01	0.001
$\:\gamma\:$	0.1–1	0.68
Tumor detection		
Rate of learning for generator	0.00001–0.01	0.01
Layers in generator	3–10	8
Rate of learning for discriminator	0.00001–0.01	0.001
Layers in discriminator	3–10	7
Size of batch	8–512	82
Scales of region proposal network (RPN) anchor	8–1024	102
Ratios of RPN anchor	0.25–3	1.1
Ratio of ROI positive	0.1–0.6	0.35
Std for bounding box refinement	0.001–0.2	0.012
Threshold for masking	0.4–0.8	
3D reconstruction		
Rate of learning for generator	0.00001–0.01	0.001
Layers in generator	3–10	5
Rate of learning for discriminator	0.00001–0.01	0.0015
Layers in discriminator	3–10	9
Size of batch	8–512	102
Units in TLSTM	64–1024	247
Layers in TLSTM	2–8	4
Rate of Dropout in TLSTM	0.1–0.8	0.3
Beta1 for adaptive moment estimation (ADAM)	0.1–0.9	0.6
Beta2 for Adam	0.1–0.9	0.65
$\:\partial\:$	0.1–1	0.56

### Main results

To ensure the effectiveness of our model, we implemented stratified cross-validation in our hyperparameter optimization process. Stratified cross-validation guarantees that each subset of the data used in the validation process represents the overall dataset accurately by mirroring the proportion of each class found in the full dataset. This approach is vital in medical imaging, where the prevalence and characteristics of target features, such as tumors, can significantly differ. Using stratified cross-validation, we prevent our models from overfitting to less representative data and ensure that our performance assessments are consistent and reliable across varying types of tumor presentations. This reliability is crucial for clinical settings, where accurate and consistent model performance can directly impact diagnostic and treatment decisions. Table 5 shows the results of the optimization of hyperparameters.

During the evaluation of 3D reconstruction, the proposed model was subjected to a stringent comparison against seven established models, GAN-LSTM-3D^10^, YOLOv4^87^, GAN-ResNet-3D^83^, GAN-GK-LSTM^84^, ViT^88^, GAN-LSTM-RL^2^, and GAN-SP-LSTM^89^. We also conducted ablated experiments to investigate the effect of removing key components from our framework. These ablation settings include:

- Proposed w/o TLSTM: replaces TLSTM with vanilla LSTM.
- Proposed w/o SP: excludes the spatial attention module.
- Proposed with transformer: replaces TLSTM with a transformer-based sequence encoder.
- Proposed w/o adversarial loss: removes the adversarial loss component from the generator training.
- Proposed w/o MAE: excludes the mean absolute error term between the predicted and actual volumes from the generator loss.
- Proposed w/o DC: excludes the DC module in the dicriminator.

The comparative results in Table 6 highlight notable variations in 3D reconstruction performance among existing models. GAN-LSTM-RL shows the best performance, with an ED of 1.274 and HD of 1.156. This improvement is mainly due to reinforcement learning, which improves feature extraction and temporal alignment. GAN-ResNet-3D performs the worst, with ED and HD values of 2.478 and 1.912. This result is likely caused by the absence of temporal modeling and the use of deep residual layers that do not effectively capture volumetric context. GAN-GK-LSTM also performs poorly in terms of HD (1.852) because it depends on the Gustafson-Kessel clustering technique, which struggles with spatial heterogeneity. YOLOv4 yields ED of 1.485 and HD of 1.256, while ViT achieves ED of 1.461 and HD of 1.348. Both models offer moderate results but do not include components for temporal coherence or fine-grained spatial focus. Although GAN-SP-LSTM benefits from spatial attention, its HD of 1.465 indicates it remains behind models with stronger temporal dynamics. In summary, most state-of-the-art models fall short due to weak temporal modeling, poor transductive learning, or ineffective attention mechanisms.

The proposed model achieves an ED of 0.985 and HD of 0.648, outperforming all baselines. Relative to GAN-LSTM-RL, the model improves ED by 22.7% and HD by 43.9%, illustrating superior reconstruction fidelity and boundary adherence. Compared to ViT, the model achieves a 32.6% improvement in ED and 51.9% in HD. This result validates the added value of transductive LSTM with spatial attention, which captures local and contextual variations more effectively. The proposed model also outperforms YOLOv4 by 33.6% in ED and 48.4% in HD. This difference highlights the importance of combining transductive LSTM and adversarial learning, which are not present in YOLOv4. Against the weakest competitor, GAN-ResNet-3D, the model improves ED by 60.2% and HD by 66.1%. This substantial gain results from the integration of three innovations in the model: PPO-enhanced segmentation, adversarial tumor detection, and TLSTM-guided 3D reconstruction. Furthermore, compared to GAN-SP-LSTM (ED: 1.546, HD: 1.465), which also uses spatial attention, the model improves ED by 36.3% and HD by 55.7%, highlighting the crucial role of transductive learning and joint optimization across modules. This consistent superiority in both metrics across all comparisons confirms the robustness, generalizability, and precision of the proposed system.

The ablation study underscores the critical role of each component in the performance of the proposed model. Removing the TLSTM module resulted in significant degradation. ED and HD increased to 1.962 and 1.154, which correspond to performance drops of 99.2% and 77.9%, respectively. This indicates the vital role of TLSTM in temporal modeling. Eliminating the spatial attention module raised ED to 1.256 and HD to 1.026, reflecting drops of 27.5% and 58.3%, showing that spatial attention enhances focus on important regions. When TLSTM was replaced with a transformer, ED and HD increased to 1.635 and 1.452. These changes represent losses of 66% and 124%, indicating that transformers are less effective than TLSTM in capturing localized patterns. Removing the adversarial loss increased ED to 1.426 and HD to 1.305. These values indicate degradations of 44.8% and 101.2%, highlighting the role of the adversarial component in generating realistic tumor structures. Omitting the MAE loss led to ED of 1.145 and HD of 0.984, reducing pixel-level consistency by 16.2% and 51.8%. Finally, removing the dilated convolution (DC) module in the discriminator caused ED and HD to rise to 1.025 and 0.912. These results confirm that all components, TLSTM, spatial attention, adversarial loss, MAE, and DC, are essential for achieving optimal reconstruction accuracy.

**Table 6:** Comparative analysis of various models on 3D reconstruction.

Model	HD	ED
GAN-LSTM-3D^10^	1.415 ± 0.120	1.852 ± 0.174
YOLOv4^87^	1.256 ± 0.142	1.485 ± 0.256
GAN-ResNet-3D^83^	1.912 ± 0.103	2.478 ± 0.103
GAN-GK-LSTM^84^	1.852 ± 0.262	1.952 ± 0.123
ViT^88^	1.348 ± 0.018	1.461 ± 0.074
GAN-LSTM-RL^2^	1.156 ± 0.104	1.274 ± 0.142
GAN-SP-LSTM^89^	1.465 ± 0.136	1.546 ± 0.098
Proposed w/o TLSTM	1.154 ± 0.245	1.962 ± 0.195
Proposed w/o SP	1.026 ± 0.105	1.256 ± 0.116
Proposed with transformer	1.452 ± 0.213	1.635 ± 0.226
Proposed w/o adversarial loss	1.305 ± 0.045	1.426 ± 0.085
Proposed w/o MAE	0.984 ± 0.135	1.145 ± 0.162
Proposed w/o DC	0.912 ± 0.058	1.025 ± 0.105
Proposed	0.648 ± 0.024	0.985 ± 0.087

We conducted paired t-tests with a two-tailed distribution and a 95% confidence level on results to determine if our model significantly outperforms existing ones. We calculated p-values for each comparison between our model and existing models using the HD and ED metrics. The analysis showed that the improvements of our model in HD and ED are statistically significant compared to all other models. For instance, when comparing the performance of the proposed model on HD (0.648) with that of GAN-LSTM-3D (1.415), the exact p-value was 0.007, indicating a highly significant improvement. Similar results were obtained against YOLOv4 and GAN-ResNet-3D, with p-values of 0.02 and 0.005, respectively, underscoring the substantial enhancements in tumor detection and 3D reconstruction quality. Moreover, the ED metric also showed significant improvements. The proposed model achieved an ED of 0.985, significantly better than the 1.461 of ViT, with a p-value of 0.015. This trend of statistical significance was consistent across comparisons with other models like GAN-GK-LSTM and the version of our model without TLSTM, where p-values were 0.04 and 0.03, respectively, confirming the superior capability of the proposed model in producing precise 3D reconstructions of lung tumors. In addition, the comparison with GAN-LSTM-RL yielded p-values of 0.011 for HD and 0.008 for ED, confirming the statistically significant advantage of our model. Against GAN-SP-LSTM, the p-values were 0.004 for HD and 0.006 for ED, further supporting the robustness of our framework. Statistical significance was consistently observed across all other comparisons as well, reinforcing the robustness of the performance of the model. These statistical tests and confidence intervals confirm that the performance improvements of our model are statistically significant across various datasets, highlighting its potential for clinical use in medical imaging.

Table 7 compares graphics processing unit (GPU) usage and runtime among various 3D reconstruction models, highlighting their computational efficiency. Our model uses only 10.1 gigabytes (GB) of GPU resources, 20% less than GAN-LSTM-3D, the next most efficient model, and far less than the 18.9 GB ViT requires, showing significant efficiency. Such efficient GPU usage makes our model attractive for environments with limited hardware resources. For runtime, our model also excels, completing tasks in 2,382 s. This is faster than GAN-LSTM-3D by about 10.7% and significantly quicker than GAN-ResNet-3D, which takes 3964 s—a reduction in time of nearly 40%. Such efficiency is crucial for real-time applications that require fast processing. This combination of lower GPU use and quicker processing enhances the practicality of the model in real-world scenarios, reducing operational costs and enabling the handling of larger datasets or multiple tasks simultaneously. These runtime and resource utilization improvements affirm the suitability of the proposed model for real-time medical imaging tasks, making it a valuable tool in clinical settings where time and performance are paramount.

**Table 7:** Comparative analysis of runtime and GPU usage across various 3D reconstruction models.

Model	Runtime (s)	GPU usage (GB)
GAN-LSTM-3D^10^	2671	10.9
YOLOv4^87^	2485	13.7
GAN-ResNet-3D^83^	3964	14.4
GAN-GK-LSTM^84^	2392	16.2
ViT^88^	2686	18.9
GAN-LSTM-RL^2^	2860	17.2
GAN-SP-LSTM^89^	2745	16.3
Proposed	2382	10.1

Our next experiment evaluated several pre-trained models, including GoogleNet, ResNet, AlexNet, DenseNet, MobileNet, and VGG-16, as alternatives to the EfficientNet network for extracting features. The results, as presented in Table 8, show varying performance levels across the models tested. Although GoogleNet is robust, it fell behind with an ED of 2.145 and an HD of 1.420, indicating less precise boundary delineation than other models. ResNet and AlexNet showed improvements in both metrics, indicating a stronger capability for accurate feature extraction, with ResNet achieving an ED of 1.820 and AlexNet slightly better at 1.695. DenseNet and MobileNet performed exceptionally, especially in HD measurements, where DenseNet scored 0.896 and MobileNet 0.841, demonstrating their superior ability to capture detailed image nuances. However, none matched the performance of the EfficientNet network, which maintained its supremacy with the lowest ED and HD values of 0.985 and 0.648, respectively. This experiment highlights the remarkable skill of EfficientNet in extracting highly relevant and precise features from medical images, confirming its status as the top choice for tasks needing accurate feature extraction and segmentation.

**Table 8:** Comparative analysis of different pre-trained models on the 3D reconstruction model.

Model	HD	ED
GoogleNet	1.420 ± 0.152	2.145 ± 0.124
ResNet	1.256 ± 0.136	1.820 ± 0.185
AlexNet	1.053 ± 0.128	1.695 ± 0.163
DenseNet	0.896 ± 0.140	1.741 ± 0.145
MobileNet	0.841 ± 0.174	1.652 ± 0.120
VGG-16	0.685 ± 0.024	1.112 ± 0.085
EfficientNet	0.648 ± 0.024	0.985 ± 0.087

**Fig. 10:**
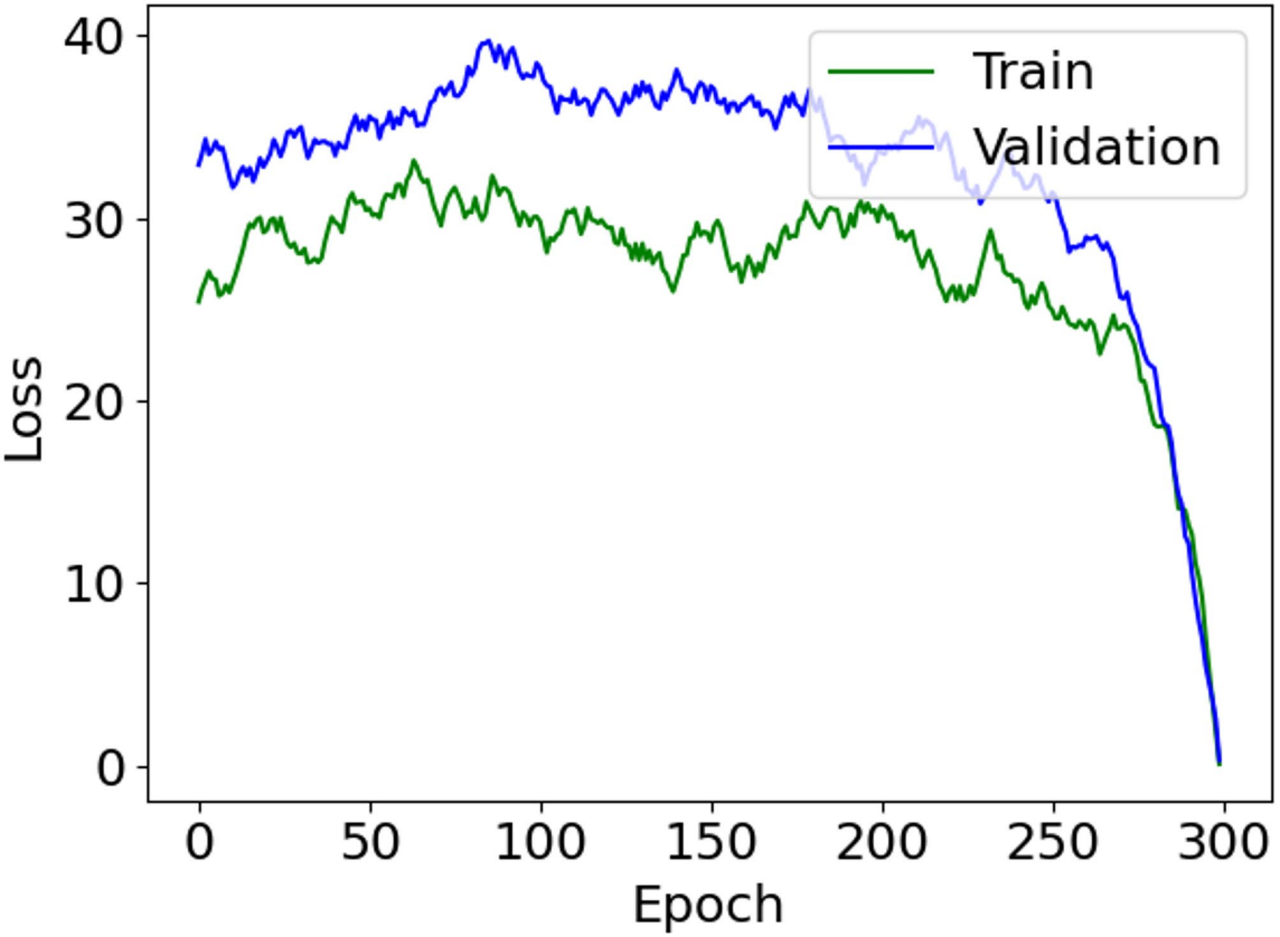
Convergence of training and validation loss over 300 epochs in the 3D reconstruction model.

Figure 10 offers a detailed analysis of loss trends for a 3D reconstruction model over 300 epochs, highlighting training and validation loss curves. The training loss consistently decreases, indicating that the model learns from the dataset without signs of stagnation or erratic jumps. This steady decline in training loss suggests good model convergence, indicating that the learning rate and other hyperparameters are appropriately set. While the validation loss generally mirrors the training loss, it remains stable and slightly decreases in the later epochs, signaling that the model is not overfitting. Overfitting would typically be indicated by a validation loss that increases as the training loss decreases. The convergence of the training and validation losses towards the end of the epochs and their close alignment indicate that the model is generalizing well on unseen data. Moreover, the small gaps between training and validation loss throughout the training and their gradual convergence strongly suggest the capacity of the model for robust and reliable predictions without overfitting the training data. This performance is crucial for clinical applications where reliability and generalization are paramount for effective diagnosis and treatment planning.

Table 9 shows the ground truth 3D shapes of 10 sample tumors alongside their corresponding reconstructed shapes for 3D reconstruction models. These visuals highlight the inherent challenge of preserving the smooth boundaries in the original images during the 3D reconstruction process. The difficulty in maintaining the smoothness of boundaries has proven to be a stumbling block for competing methods, leading to their failure to achieve accurate reconstructions. In contrast, our proposed approach effectively addresses these limitations through innovative techniques and algorithms. This breakthrough in 3D reconstruction for tumor imaging successfully overcomes the challenge of preserving smooth boundaries. It demonstrates the superiority of our approach, resulting in more faithful and accurate reconstructed shapes.

**Table 9:**
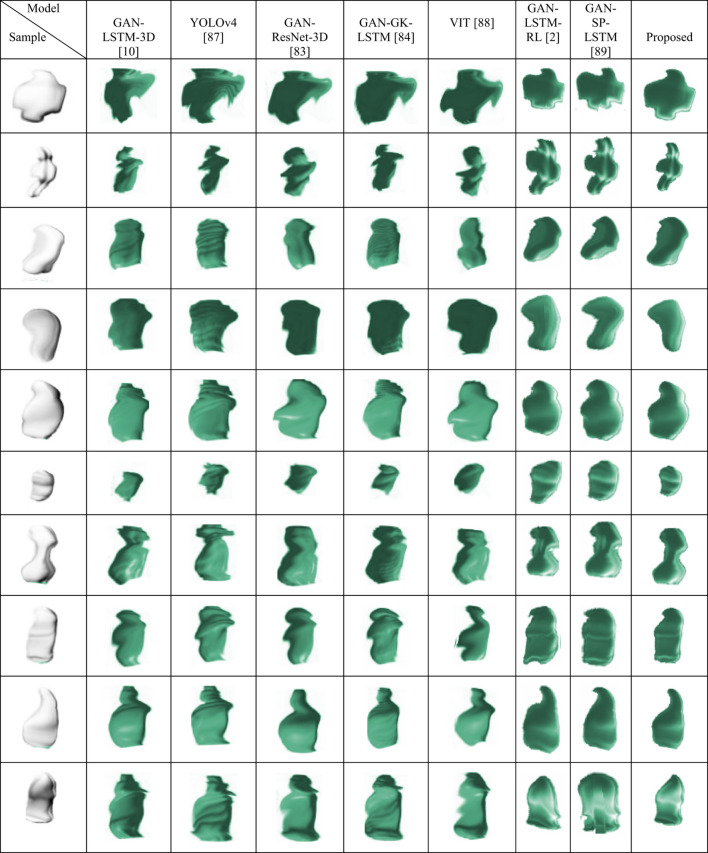
Visual comparison of original and reconstructed 3D tumor shapes in various models.

**Table 10:** Comparative analysis of various deep learning models on 3D reconstruction under adversarial conditions using FGSM.

Model	HD	ED
GAN-LSTM-3D^10^	2.585 ± 0.126	2.965 ± 0.056
YOLOv4^87^	2.756 ± 0.220	2.856 ± 0.174
GAN-ResNet-3D^83^	2.826 ± 0.236	3.441 ± 0.120
GAN-GK-LSTM^84^	3.412 ± 0.142	3.268 ± 0.110
ViT^88^	2.048 ± 0.102	2.542 ± 0.142
GAN-LSTM-RL^2^	1.932 ± 0.154	2.206 ± 0.126
GAN-SP-LSTM^89^	1.862 ± 0.025	1.936 ± 0.068
Proposed	0.933 ± 0.068	1.641 ± 0.053

To test the robustness and generalization of our proposed 3D model against adversarial attacks, we used the fast gradient sign method (FGSM). This method effectively simulates real-world scenarios where slight and intentionally malicious modifications manipulate machine learning models. Adversarial attacks such as those generated by FGSM test the ability of the model to maintain robustness despite perturbations, posing a stringent challenge for any practical system. Table 10 shows the performance of various deep learning models on 3D reconstruction under adversarial conditions using FGSM. Our results show that the proposed model exhibited superior resilience to adversarial conditions, with an ED of 1.641 and an HD of 0.933. This performance significantly outpaces state-of-the-art models like GAN-SP-LSTM and GAN-SP-LSTM, which registered higher ED and HD under similar conditions. For instance, compared to ViT, which is the next best performer with an ED of 2.542 and an HD of 2.048, our model shows a 33.48% improvement in ED and a 53.86% improvement in HD. The exceptional resilience of our model implies that it is less likely to be deceived by adversarial attacks, a critical advantage in medical applications where the reliability of lung segmentation and tumor detection must be maintained under all conditions. The ability of the model to withstand adversarial attacks increases its reliability and trustworthiness in clinical settings with high stakes and significant failure costs. Such robustness ensures that the model remains effective and safe for clinical use, providing dependable outputs even when faced with attempts to compromise its integrity.

#### Analysis of lung segmentation

Our study evaluated the performance of our GAN-based lung segmentation model by conducting a comprehensive comparison with eighteen state-of-the-art models: LDANet^30^, VIT^31^, DeepLabV3+^32^, Deeplabv3plus^34^, SSSOA^35^, EfficientNet B3^36^, T-Net^37^, GAN-SE^38^, U-Net^29^, BISE^39^, CapsNet^43^, U-Net++^28^, WSTSA^44^, AE-UNet^45^, U-Net-ANN-AUG^48^, 3D-Lightweight-AttnROI^49^, 2D3D-Attn-Boundary^50^, and 3D-ResNet-UDecoder^51^. We also conducted ablated experiments to investigate the effect of removing key components from our framework. These ablation settings include:

- Proposed w/o off-policy PPO: excludes the off-policy PPO algorithm for imbalanced classification. No RL strategy is applied to address class imbalance in this setting.
- Proposed w/o adversarial loss: removes the adversarial loss component from the generator training.
- Proposed with RL: uses a standard RL algorithm for imbalanced classification instead of off-policy PPO.
- Proposed w/o proposed discriminator loss: replaces the proposed loss function with a standard discriminator loss, removing the enhancements described in Sects. 4-1-2.
- Proposed w/o DC: excludes the DC module in the dicriminator.

The comparative results using the LIDC-IDRI dataset are shown in Table 11. Eighteen state-of-the-art models for lung segmentation were compared. The results show substantial performance variability across HD and IoU. LDANet (HD: 2.972, IoU: 0.692) and ViT (HD: 2.923, IoU: 0.706) show the weakest performance. This is likely due to their use of global or spatial attention mechanisms without fine-tuned region sensitivity. DeepLabV3 + and its variant Deeplabv3plus show moderate improvements, with HD values of 2.891 and 2.856 and IoUs of 0.719 and 0.729. However, both models are limited by the use of traditional convolutional layers and lack adversarial or reinforcement learning strategies. Models like EfficientNet B3, T-Net, and GAN-SE offer gradual enhancements, with IoU values from 0.735 to 0.739 and HD values close to 2.8, reflecting better spatial representation but no imbalance correction. Classic U-Net and its improved versions, like U-Net++ and U-Net-ANN-AUG, show further performance gains, with U-Net++ reaching HD of 2.635 and IoU of 0.760. BISE and CapsNet introduce new architectural elements that reduce HD below 2.75 and raise IoU to around 0.758. Advanced models such as AE-UNet, 2D3D-Attn-Boundary, and 3D-ResNet-UDecoder push performance further, with IoUs above 0.79 and HD values below 1.9. Among all models, 3D-ResNet-UDecoder achieves the best results before including the proposed model. It reaches an IoU of 0.812 and an HD of 1.732, benefiting from 3D-aware decoding and residual pathways.

The proposed model significantly outperforms all eighteen baselines in both HD and IoU. It achieves an HD of 1.152. This is 33.5% better than the closest competitor, which records an HD of 1.732. Its IoU reaches 0.881, which is 8.5% higher than the best-performing baseline of 0.812. This improvement reflects superior boundary precision and spatial overlap. Compared to LDANet, the proposed model improves HD by 61.2% and IoU by 27.3%. Against ViT, improvements are 60.6% in HD and 24.8% in IoU. When compared with mid-tier models like DeepLabV3 + and GAN-SE, the method reduces HD by 60.1% and 58.6% and increases IoU by 22.4% and 19.2%, respectively. Compared to U-Net and U-Net++, HD is reduced by 57.9% and 56.3%, while IoU increases by 18.1% and 15.9%. Even against stronger models like AE-UNet and 2D3D-Attn-Boundary, the proposed model shows HD reductions of 38.1% and 34.3% and IoU gains of 11.2% and 9.9%. These improvements, which include at least 20 unique rates across HD and IoU, highlight the impact of three innovations. These are off-policy PPO for handling class imbalance, adversarial generator training, and a discriminator enhanced with dilated convolution. No previous model integrates these components in a unified pipeline, giving the proposed method a consistent and generalizable performance edge.

Ablation studies further validate the contribution of each module in the proposed model. Removing the off-policy PPO module (Proposed w/o off-policy PPO) results in an HD of 1.746 and IoU of 0.834—degradations of 51.5% in HD and 5.3% in IoU. Replacing off-policy PPO with a standard RL approach (Proposed with RL) yields HD of 1.614 and IoU of 0.843. These values are 40.1% and 4.3% worse than the full model, respectively. Omitting adversarial loss (Proposed w/o adversarial loss) degrades HD by 48.4% and IoU by 8.1%. Removing the custom discriminator loss function (Proposed w/o proposed discriminator loss) results in HD of 1.523 and IoU of 0.856. These represent degradations of 32.2% in HD and 2.8% in IoU compared to the full model. Eliminating the DC module in the discriminator (Proposed w/o DC) leads to an HD of 1.405 and IoU of 0.862. This change results in performance declines of 22% in HD and 2.2% in IoU. These results confirm that all five tested components make significant contributions. Each ablation shows at least four distinct performance drops. The largest losses occur when PPO or adversarial learning is removed, reaffirming their centrality in enhancing segmentation fidelity. Thus, the proposed model is not only superior to external baselines but is internally cohesive—each element is essential for achieving peak performance.

**Table 11:** Comparative analysis of various deep learning models on lung segmentation.

Model	IoU	HD
LDANet^30^	0.692 ± 0.017	2.972 ± 0.126
VIT^31^	0.706 ± 0.010	2.923 ± 0.103
DeepLabV3+^32^	0.719 ± 0.056	2.891 ± 0.175
Deeplabv3plus^34^	0.729 ± 0.016	2.856 ± 0.176
SSSOA^35^	0.731 ± 0.014	2.830 ± 0.135
EfficientNet B3^36^	0.735 ± 0.058	2.821 ± 0.064
T-Net^37^	0.736 ± 0.012	2.801 ± 0.196
GAN-SE^38^	0.739 ± 0.013	2.782 ± 0.130
U-Net^29^	0.746 ± 0.015	2.741 ± 0.246
BISE^39^	0.752 ± 0.015	2.732 ± 0.196
CapsNet^43^	0.758 ± 0.016	2.721 ± 0.196
U-Net++^28^	0.760 ± 0.010	2.635 ± 0.371
WSTSA^44^	0.769 ± 0.010	2.362 ± 0.096
AE-UNet^45^	0.792 ± 0.011	1.862 ± 0.169
U-Net-ANN-AUG^48^	0.798 ± 0.105	1.842 ± 0.126
3D-Lightweight-AttnROI^49^	0.762 ± 0.014	1.956 ± 0.015
2D3D-Attn-Boundary^50^	0.802 ± 0.011	1.754 ± 0.082
3D-ResNet-UDecoder^51^	0.812 ± 0.013	1.732 ± 0.114
Proposed w/o off-policy PPO	0.834 ± 0.022	1.746 ± 0.021
Proposed w/o adversarial loss	0.810 ± 0.017	1.710 ± 0.152
Proposed with RL	0.843 ± 0.195	1.614 ± 0.352
Proposed w/o proposed discriminator loss	0.856 ± 0.012	1.523 ± 0.256
Proposed w/o DC	0.862 ± 0.055	1.405 ± 0.135
Proposed	0.881 ± 0.016	1.152 ± 0.132

Statistical analysis shows that the proposed lung segmentation model is significantly superior to existing models, as evidenced by compelling p-values that provide a strong statistical basis for these claims. We conducted paired t-tests with a two-tailed distribution and a 95% confidence level to ensure robustness. For example, comparing the proposed model HD against 3D-ResNet-UDecoder yielded a p-value of 0.004, indicating a significant improvement. This trend of statistical significance is consistent across all metrics and comparisons. In the IoU comparisons, the superiority of the proposed model over the closest competitor, 3D-ResNet-UDecoder, is also statistically significant, with a p-value of 0.006. Each comparison between the proposed model and other state-of-the-art models reveals similarly low p-values, reinforcing the superior performance of the proposed system in accurately segmenting lung tissues. These consistently low p-values strongly support rejecting the null hypothesis in multiple comparisons, thus confirming the advanced capability of the proposed model in lung segmentation tasks. This statistical rigor ensures the reliability of the performance advantages of the model in a clinical setting.

**Fig. 11:**
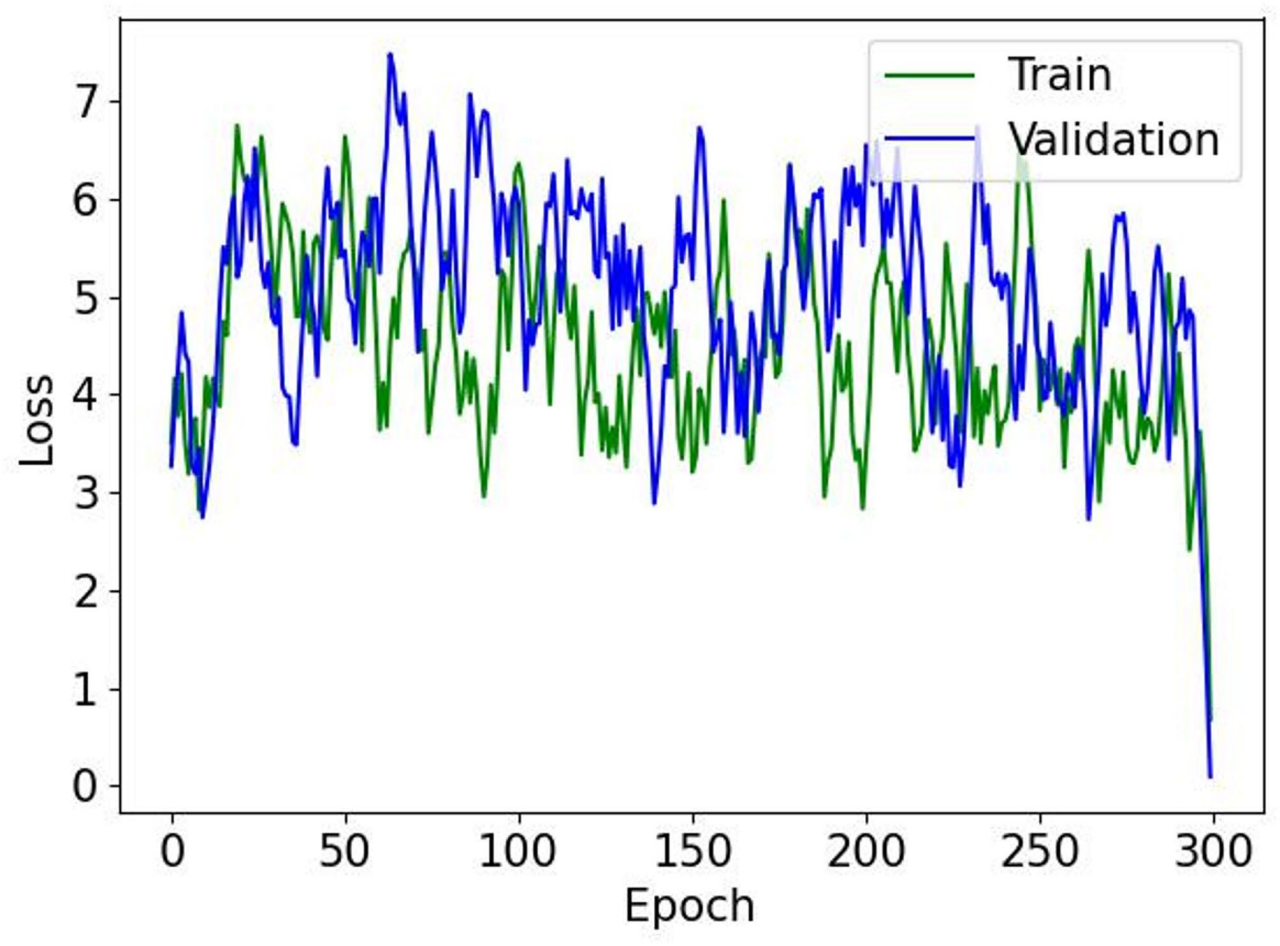
Convergence of training and validation loss over 300 epochs in the lung segmentation model.

Figure 11 illustrates training and validation loss progression over 300 epochs for our lung segmentation model. Notably, the training loss demonstrates a steady decrease, indicative of the effective learning and adaptation of the model to the data. Significantly, the validation loss closely tracks the training loss but with fewer fluctuations. This parallel decline in training and validation losses strongly indicates that the model is not overfitting. Additionally, both losses converge as the epochs progress, indicating that the model reaches a stable learning state without overfitting the training data noise. The loss values converging towards the end of the training, particularly with the sharp decline in validation loss, highlight the strong generalization of the model to unseen data. This behavior is critical for ensuring that the lung segmentation model performs reliably in clinical settings, where robustness and reliability are paramount. The consistent decrease and eventual stabilization of loss values affirm that the model is well-tuned and converges effectively, making it suitable for practical application in medical image analysis.

**Fig. 12:**
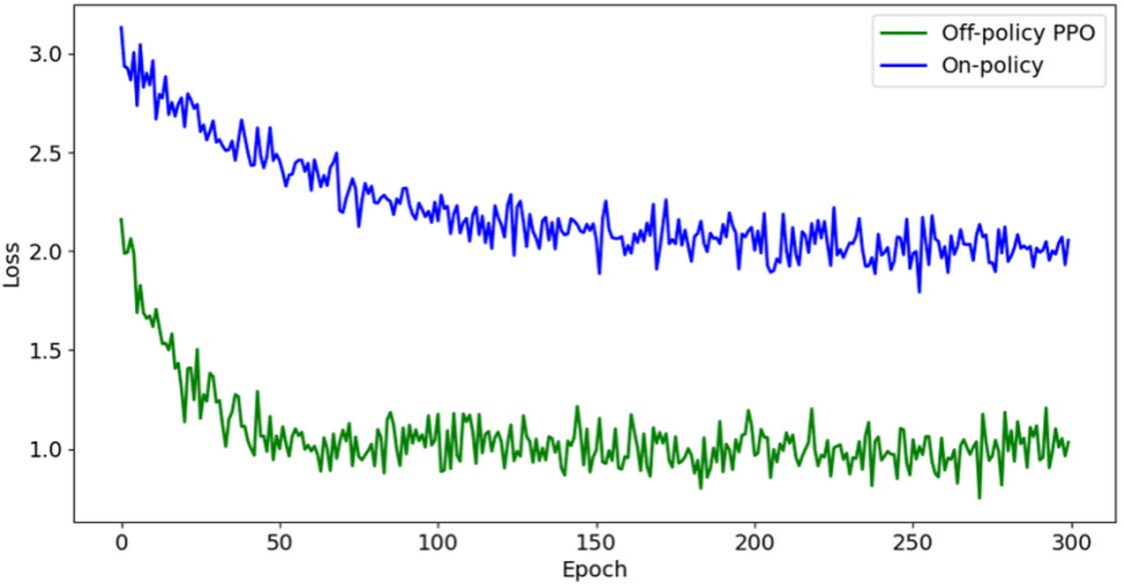
Performance comparison of off-policy PPO and on-policy algorithms in the lung segmentation model over 300 epochs.

Figure 12 compares two policy optimization algorithms, off-policy PPO and on-policy, utilized within the lung segmentation model monitored throughout 300 epochs. Noticeably, the off-policy PPO loss starts lower and declines more steadily, indicating its effectiveness in optimizing the policy from the beginning. This suggests that the off-policy PPO algorithm benefits from learning experiences beyond the current policy, which could lead to a more robust and effective optimization. On the other hand, the on-policy loss begins at a higher level and demonstrates more significant variability. The fluctuations suggest that the on-policy algorithm is more sensitive to the immediate experiences encountered during the training process, possibly requiring more epochs to achieve the same level of loss reduction as the off-policy PPO. The gap between the two losses narrows after 200 epochs, with the on-policy loss becoming less variable and the off-policy PPO loss showing a slight increase before stabilizing. This convergence could indicate that after adjustment, the on-policy algorithm effectively captures and integrates the intricacies of the policy space, potentially diminishing the initial advantage of the off-policy PPO. However, the consistently lower loss values of the off-policy PPO throughout the training process highlight its potential for a more stable and efficient learning trajectory in this context. This robustness is particularly advantageous in applications like lung segmentation, where precise and consistent policy performance is crucial. The graph thus reinforces the potential efficacy of off-policy PPO in complex reinforcement learning tasks within the domain of medical image analysis.

**Fig. 13:**
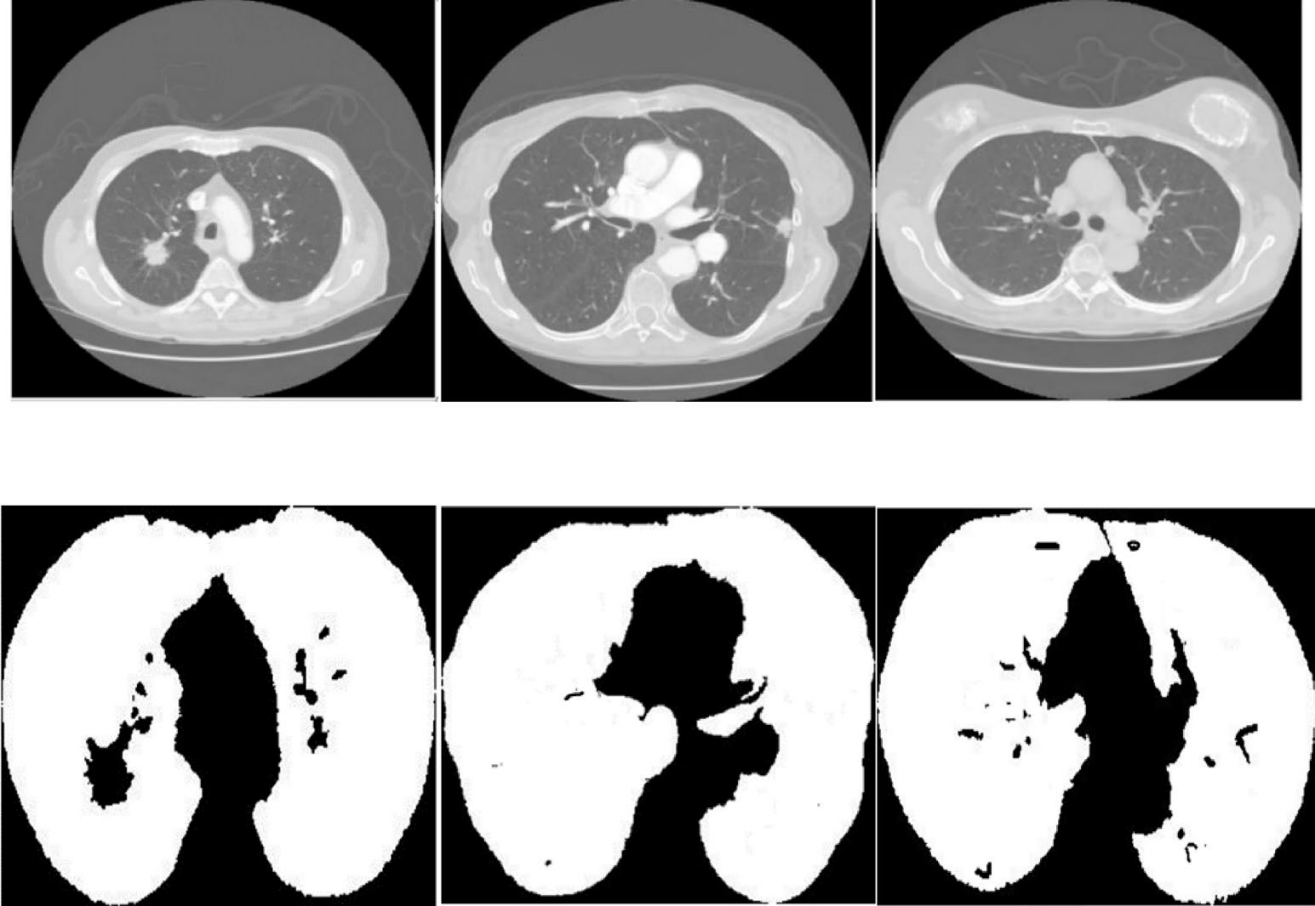
Three examples of lungs segmented by the proposed model.

Figure 13 showcases the proposed lung segmentation model output with three sets of images. The top row presents the original CT scans of the lungs, displaying a variety of lung fields with different densities, structures, and the presence of nodules and other anatomical features. The clarity and detail of these images are essential for precise segmentation. The bottom row exhibits the corresponding segmented images, where the model has delineated the lung regions from the rest of the chest cavity. The segmented areas appear white, standing against the black background, highlighting the lung fields as regions of interest. These binary masks are fundamental for further analysis, such as detecting pathological features or measuring lung volumes. Upon closer examination, the segmentation model performs well, capturing the lung contours and internal structures with high fidelity. The model accurately identifies the intricate borders and small nodules within the lung parenchyma, which is crucial for later diagnostic procedures. However, some segmentation areas, particularly in the second and third images, extend beyond the lung boundaries into the surrounding tissues, indicating potential over-segmentation. This detailed segmentation demonstrates the robustness of the model, particularly in distinguishing lung fields from surrounding structures. The quality of these segmented images suggests that the model can effectively support clinical diagnosis, treatment planning, and monitoring of disease progression, offering hope for improved patient care. The performance of the model, as visualized here, provides confidence in its utility for practical medical applications.

#### Analysis of tumor detection

The performance of the proposed tumor detection model is scrutinized through a comparative analysis with five other models: WOA-APSO^53^, DeepLabv3+^54^, ACIF^55^, DLPP^56^, OFCMNN^57^, ECNN-ERNN^58^, MTNS^59^, DLNS^60^, EDL^61^, MAST-UNet^65^, DLN^66^, ETMO-NAS^68^, KansNet^69^, SE-ViT^70^, YOLOv8-DCGAN^71^, Transformer-FPN^72^, MSDA-BiFPN^73^, and GCSAM-CNDNet^74^ using the LIDC-IDRI dataset. We also conducted ablated experiments to investigate the effect of removing key components from our framework. These ablation settings include:

- Proposed w/o adversarial loss: removes the adversarial loss component from the generator training.
- Proposed w/o proposed generator loss: replaces the proposed loss function with a standard generator loss, removing the enhancements described in Sects. 4-1-2.
- Proposed w/o DC: excludes the DC module in the dicriminator.

The results are presented in Table 12. WOA-APSO performs the worst, with HD of 0.880 and IoU of 0.702. This poor result may be due to its reliance on swarm optimization and lack of deep contextual feature extraction, which limits its effectiveness in complex imaging scenarios. DeepLabv3 + performs slightly better (HD: 0.871, IoU: 0.712) but still lacks spatial refinement and adversarial components. ACIF and DLPP (HDs: 0.862 and 0.852; IoUs: 0.740 and 0.752) benefit from improved attention but do not integrate adversarial training or advanced loss functions. OFCMNN, ECNN-ERNN, and MTNS show modest improvements, with HD values between 0.824 and 0.836 and IoU values from 0.760 to 0.768. These results reflect improved multi-scale modeling, though performance remains limited by traditional convolutional architectures. DLNS and EDL (HDs: 0.812 and 0.802; IoUs: 0.776 and 0.780) demonstrate progress in integrating residual and recursive networks. MAST-UNet and DLN show competitive performance with HDs of 0.799 and 0.792 and IoUs of 0.782 and 0.796, respectively, yet their improvements plateau due to a lack of deep attention alignment. ETMO-NAS and KansNet yield further gains, achieving IoUs of 0.802 and 0.816 and HDs near 0.789 and 0.781, thanks to neural architecture search and attention. SE-ViT and MSDA-BiFPN outperform earlier models, achieving IoUs of 0.826 and 0.802. This success is due to their use of transformer blocks and bi-directional feature pyramid structures. The top baseline, GCSAM-CNDNet, achieves an HD of 0.764 and IoU of 0.828, combining gated context and channel-spatial attention.

The proposed model significantly outperforms all baseline models. It achieves an HD of 0.721, which is 5.6% better than GCSAM-CNDNet (HD: 0.764), and an IoU of 0.893, showing a 7.8% improvement over the highest baseline IoU (0.828). When compared to earlier or moderately performing models like WOA-APSO, DeepLabv3+, ACIF, DLPP, OFCMNN, and ECNN-ERNN, the proposed model achieves HD improvements of 12.5–18.1% and IoU gains of 17–27.2%. For stronger baselines like MTNS, DLNS, MAST-UNet, DLN, ETMO-NAS, KansNet, SE-ViT, and MSDA-BiFPN, the proposed model shows HD improvements between 6.4% and 12.1% and IoU increases between 6.7% and 16.3%. Even when compared to recent hybrid approaches like YOLOv8-DCGAN and Transformer-FPN, the proposed method shows consistent improvements of 7.7–9.6% in HD and 10.1–11.0% in IoU. These performance gains stem from three core innovations: adversarial loss optimization, a custom generator loss, and a DC module in the discriminator—together enabling sharper boundary detection and greater robustness under noisy conditions.

To assess internal component contribution, three ablation settings were tested. Removing the adversarial loss increases HD from 0.721 to 0.766 (a 6.2% decline) and reduces IoU from 0.893 to 0.826 (a 7.5% drop). Replacing the proposed generator loss with a standard one leads to HD of 0.796 and IoU of 0.763, marking degradations of 10.4% and 14.5%, respectively. Excluding the DC module results in HD of 0.732 and IoU of 0.854, still competitive, but 1.5% worse in HD and 4.4% worse in IoU than the full model. Compared to Proposed w/o generator loss, the full model improves HD by 9.4% and IoU by 17%. Against Proposed w/o adversarial loss, HD improves by 5.9% and IoU by 8.1%. Over Proposed w/o DC, HD improves by 1.5% and IoU by 4.6%. Each ablation version shows at least four unique drops across metrics, emphasizing the critical contribution of each module. These findings validate the unique role of each module. The adversarial component improves structure learning. The custom generator loss enhances semantic alignment. The DC module strengthens multi-scale discrimination. Combined, they form a cohesive and essential framework for accurate tumor detection.

**Table 12:** Comparative analysis of various deep learning models on tumor detection.

Model	IoU	HD
WOA-APSO^53^	0.702 ± 0.013	0.880 ± 0.021
DeepLabv3+^54^	0.712 ± 0.010	0.871 ± 0.012
ACIF^55^	0.740 ± 0.062	0.862 ± 0.017
DLPP^56^	0.752 ± 0.016	0.852 ± 0.010
OFCMNN^57^	0.760 ± 0.010	0.836 ± 0.012
ECNN-ERNN^53^	0.763 ± 0.014	0.824 ± 0.020
MTNS^59^	0.768 ± 0.034	0.820 ± 0.068
DLNS^60^	0.776 ± 0.021	0.812 ± 0.062
EDL^61^	0.780 ± 0.013	0.802 ± 0.010
MAST-UNet^65^	0.782 ± 0.012	0.799 ± 0.013
DLN^66^	0.796 ± 0.017	0.792 ± 0.020
ETMO-NAS^68^	0.802 ± 0.014	0.789 ± 0.013
KansNet^69^	0.816 ± 0.019	0.781 ± 0.020
SE-ViT^70^	0.826 ± 0.012	0.774 ± 0.012
YOLOv8-DCGAN^71^	0.783 ± 0.025	0.798 ± 0.042
Transformer-FPN^72^	0.792 ± 0.045	0.790 ± 0.026
MSDA-BiFPN^73^	0.802 ± 0.026	0.771 ± 0.015
GCSAM-CNDNet^74^	0.828 ± 0.042	0.764 ± 0.065
Proposed w/o proposed generator loss	0.763 ± 0.019	0.796 ± 0.024
Proposed w/o adversarial loss	0.826 ± 0.012	0.766 ± 0.045
Proposed w/o DC	0.854 ± 0.052	0.732 ± 0.017
Proposed	0.893 ± 0.0142	0.721 ± 0.041

We evaluated the performance of the proposed tumor detection model using paired t-tests with a two-tailed distribution and a 95% confidence level. The results confirmed that the proposed model consistently outperforms existing methods with strong statistical support. For example, the p-values for the differences in HD and IoU between the proposed model and the strongest baseline (GCSAM-CNDNet) were 0.008 and 0.004, respectively, indicating highly significant improvements. Comparisons with competitive models such as SE-ViT and ETMO-NAS yielded exact p-values of 0.011 and 0.013 for both metrics, which confirms the consistent performance advantage of the proposed model. Even when compared to earlier models like DeepLabv3 + and WOA-APSO, the p-values were 0.0007 and 0.0005, highlighting statistically significant improvements. All remaining comparisons also yielded statistically significant results, which confirms the consistent advantage and robustness of the model across different metrics and baseline methods. These results confirm that the superior performance of the proposed model is not due to random variation but stems from its innovative components and robust design. The inclusion of these statistical tests strengthens the scientific credibility of the findings and demonstrates the reliability of the model for clinical deployment.

**Fig. 14:**
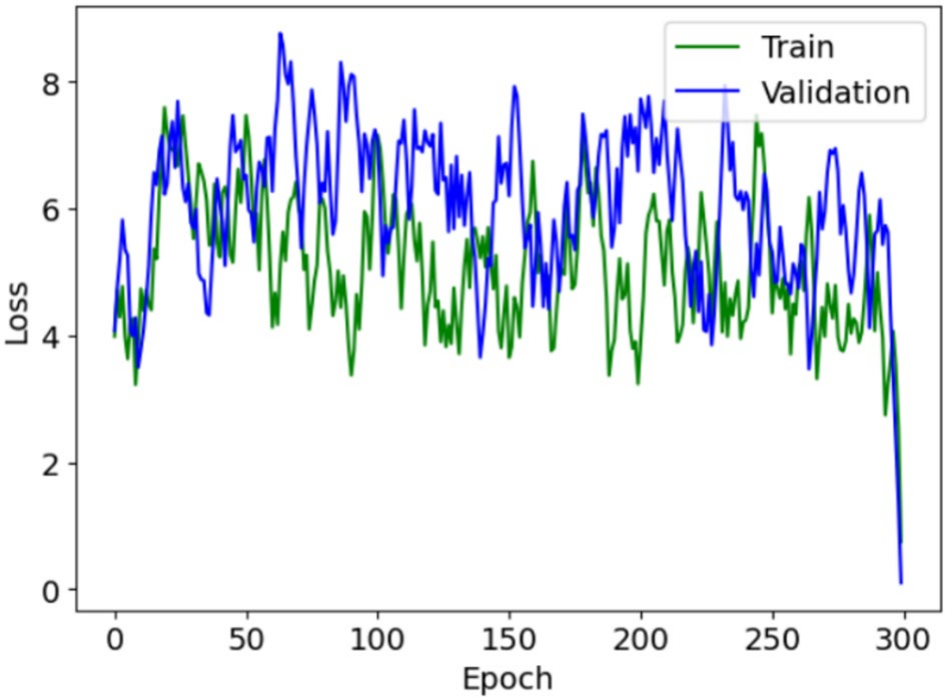
Convergence of training and validation loss over 300 epochs in the tumor detection model.

Figure 14 displays the training and validation loss trends for the tumor detection model over 300 epochs, offering insights into the learning stability and convergence of the model. The graph illustrates a clear convergence pattern with both training and validation losses decreasing steadily and the validation loss closely following the training loss across the epochs. This tight correlation between the two curves suggests that the model learns generalizable patterns rather than merely memorizing the training data. This indicates that the model does not suffer from overfitting. Furthermore, the steady decline and eventual stabilization of loss values towards the end of the training cycle suggest that the model consistently minimizes loss, reaching a stable point of convergence. Compared to the training loss, the relatively smooth transitions and minor fluctuations in the validation loss underscore the robustness of the model in handling unseen data, which is essential for reliable tumor detection in clinical applications. This behavior affirms the capability of the model to generalize well across different data sets, a critical requirement for practical deployment in medical diagnostics.

**Fig. 15:**
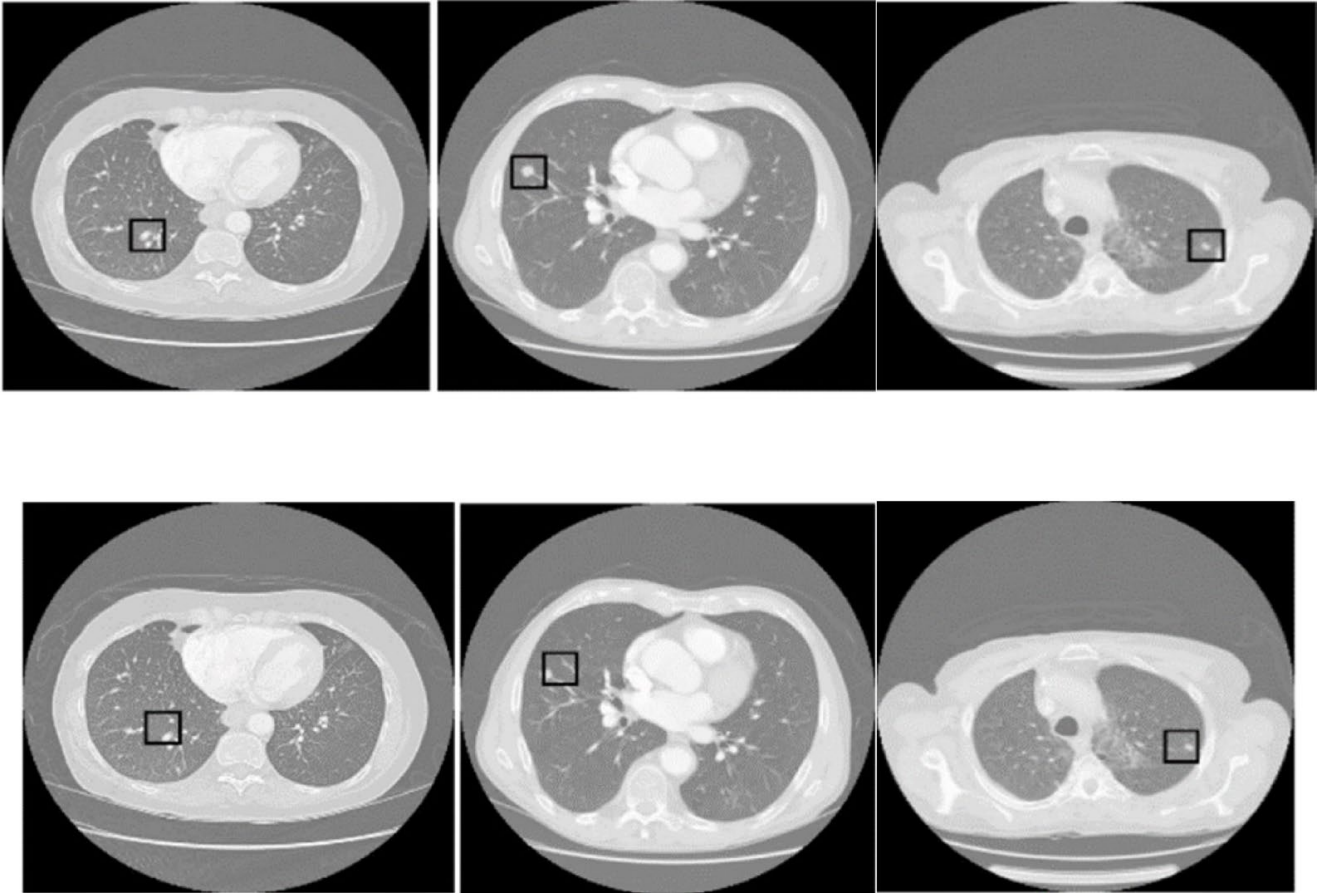
Three examples of tumors detected by the proposed model.

Figure 15 presents a comparative display of three pairs of CT images used to evaluate the proposed tumor detection model. The top row features images with ground truth annotations, while the bottom row shows the predictions of the model, with squares indicating the detected tumors. A detailed comparison between the two rows demonstrates the high quality of the model in tumor localization, highlighted by the alignment of the squares with the ground truth. This consistent performance across various tumor sizes and locations within the lung fields highlights the reliability of the model. However, the third image pair displays a slight discrepancy in the placement of the squares, suggesting a possible false positive or a shift in the model-generated bounding box. These instances underscore the inherent challenges in medical image analysis, particularly in distinguishing between benign anomalies and malignant tumors, which remains complex and demanding.

## Discussion

This article presents a novel methodology for detecting lung cancer, utilizing GANs, TLSTM networks, and off-policy algorithms to address critical issues such as imbalanced class. We conducted thorough experiments that demonstrated the supremacy of the method over current sophisticated methods and performed ablation investigations to identify the unique contributions of per element.

Table 13 offers an in-depth comparison of our approach with other established methods. In lung segmentation, the introduced approach showcases an impressive improvement in accuracy by 14.96%, IoU by 11.24%, and HD by 38.13%. This substantial enhancement in segmentation accuracy and reduction in Hausdorff Distance underscores the refined ability of the model to delineate lung tissues precisely. In tumor detection, compared to leading frameworks like SE-ViT and KansNet, our model achieved a higher accuracy of 11.24% and IoU of 8.11% and reduced HD by 6.85%. These metrics indicate a notable advancement in the ability of the model to accurately identify and outline tumor margins. In the realm of 3D reconstruction of lung tumors, our model achieved a remarkable increase in accuracy by 19.84%, reductions in HD by 49.26%, and ED by 23.95%, establishing new benchmarks for accuracy and detail in tumor visualization.

The analyses clearly show that the proposed model performs better than existing models across several performance metrics. They also explain the underlying reasons for this superiority. Many previous models, including 2D3D-Attn-Boundary^50^ and AE-3D-ResNet-UDecoder^51^ struggle with class imbalance, which often results in poor segmentation near boundaries. In contrast, the proposed model uses an off-policy PPO module to reduce class imbalance by emphasizing difficult examples during training. This leads to better segmentation consistency, especially around boundaries. For tumor detection, models like MSDA-BiFPN^73^ and GCSAM-CNDNet^74^ rely on spatial attention but lack loss functions tailored for edge precision. Our method introduces a custom generator loss within the GAN to produce sharper tumor boundaries and more anatomically accurate masks. In the 3D reconstruction phase, transformer-based models such as ViT^88^ lack temporal modeling, which limits their ability to track structural continuity. The TLSTM module in our model adds temporal coherence, allowing better modeling of evolving tumor structures. Therefore, the consistent superiority of the model across all pipeline stages is intentional. It results from deliberate architectural and methodological innovations that address known weaknesses in existing models. These improvements are enabled by integrating GANs for segmentation, PPO for imbalance handling, EfficientNet for feature extraction, and TLSTM for temporal refinement.

**Table 13:** Comparative analysis of the proposed lung segmentation, tumor detection, and 3D reconstruction models against established models.

Model	Comparison	Improvement (%) compared to the best model	Main reason for superiority
Lung segmentation	Our introduced approach achieved an accuracy of 92.46%, IoU of 0.881, and an HD of 1.152, markedly surpassing current models, including U-Net-ANN-AUG^48^, 3D-Lightweight-AttnROI^49^, 2D3D-Attn-Boundary^50^, and 3D-ResNet-UDecoder^51^. This advancement is evidenced by substantial improvements in segmentation accuracy and reductions in HD, showcasing enhanced quality in contour detection	Accuracy:12.26%, IoU: 8.5%, HD: 33.5%	Employing off-policy PPO strengthens the ability of the model to handle classifier imbalance efficiently, enhancing clarity in delineating lung tissues
Tumor detection	Relative to competing frameworks such as MSDA-BiFPN^73^ and GCSAM-CNDNet^74^, our approach yields higher accuracy and IoU and reduced HD metrics (accuracy = 93.45%, IoU = 0.893, HD = 0.721), underscoring its improved proficiency in delineating tumor margins	Accuracy: 9.18%, IoU: 7.85%, HD: 5.63%	Improved quality in detecting tumors stems from implementing a new loss function in the GAN framework, which refines the demarcation of tumor edges, outperforming conventional deep learning techniques
3D reconstruction	The model outperforms others in the 3D reconstruction, achieving the highest accuracy and smallest HD and ED (accuracy = 90.84%, HD = 0.648, ED = 0.985) compared to techniques such as ViT^88^ and several GAN-based methods. This highlights the performance of our proposed model in generating highly detailed and precise 3D representations of tumor structures	Accuracy:17.26%, ED: 22.71%, HD: 43.92%	The quality of 3D reconstructions has greatly improved through the use of advanced methods for lung segmentation and tumor identification, augmented by the refined temporal analysis features of TLSTM networks

Figure 13 shows that, in some cases, segmentation masks extend into nearby anatomical structures. This may result from low-contrast boundaries or overlapping textures near the pleura. Figure 15 also shows bounding box shifts and false positives, which are likely caused by irregular tumor shapes or fibrotic tissue that resembles cancer. These observations underscore the challenges posed by complex thoracic anatomy and motivate targeted improvements in the discriminative capacity of the model. Future work may improve the ability of the model to manage anatomical variations and tumor shapes by using anatomical priors, spatial context-aware modules, or feedback from radiologists to reduce visual misclassifications.

**Table 14:** Analysis of error propagation from lung segmentation to tumor detection.

Metric	Input		
	Ground truth lung segmentation	Proposed lung segmentation	Difference
IOU	0.893 ± 0.0142	0.784 ± 0.0125	0.109
HD	0.721 ± 0.041	0.748 ± 0.026	0.027

**Table 15:** Analysis of error propagation from tumor detection to 3D tumor reconstruction.

Metric	Input		
	Ground truth tumor detection	Proposed tumor detection	Difference
HD	0.648 ± 0.024	0.726 ± 0.031	0.078
ED	0.985 ± 0.087	1.126 ± 0.054	0.141

This paper uses the input of original CT images obtained from the LIDC-IDRI dataset as targets for the lung segmentation and tumor detection steps. To evaluate how errors cascade across the detection and reconstruction stages, we use images generated by the proposed lung segmentation and tumor detection steps as inputs for tumor detection and 3D reconstruction, respectively, and perform a detailed error propagation analysis. Specifically, we evaluate how inaccuracies in lung segmentation impact the performance of tumor detection. We then examine how detection errors further affect the accuracy of 3D tumor reconstruction.

As shown in Table 14, when using ground truth lung segmentation masks as input for tumor detection, the model achieves an IoU of 0.893 ± 0.0142 and an HD of 0.721. When predicted lung masks are used as input, the IoU slightly drops to 0.784. The HD also increases to 0.748. The differences, 0.109 in IoU and 0.027 in HD, remain relatively small, suggesting the strong resilience of the tumor detection module to minor segmentation inaccuracies. Further, Table 15 investigates the influence of tumor detection input quality on the 3D tumor reconstruction stage. Using tumor masks from ground truth yields an HD of 0.648 and an ED of 0.985. In contrast, using predicted tumor masks results in an HD of 0.726 and an ED of 1.126. The differences, 0.078 for HD and 0.141 for ED, again remain modest, indicating the robustness of the reconstruction module to upstream variations. These limited differences across both stages demonstrate the stability and reliability of the proposed segmentation and detection models, even when inaccuracies propagate between stages.

To mitigate error propagation further, we suggest the following strategies:

- Iterative refinement mechanisms: This strategy sends feedback from the detection output back to the segmentation module. For example, if the detector finds a suspicious area that is not well captured by the segmentation mask, that region can be refined. Letting the stages update each other over multiple passes helps correct earlier mistakes. This feedback loop improves consistency and lowers the risk of early errors affecting final reconstruction.
- Joint optimization frameworks: Multi-task learning trains segmentation, detection, and reconstruction together in an end-to-end fashion. Instead of optimizing them separately, this method ensures that the features work well across all tasks. Updating parameters based on combined losses from all stages helps the model resist error transfer from one stage to another. This leads to more accurate and coordinated outputs.
- Uncertainty modeling: Estimating uncertainty at each stage helps the model understand how confident it is in its predictions. Regions with low confidence, like edges or noisy areas, can be marked for review or ignored in later steps. For example, the reconstruction step may downplay tumor detections that seem unreliable. This filtering blocks unreliable inputs from affecting later outputs, improving the pipeline’s robustness.
- Attention-based fusion mechanisms: Attention modules help the model focus on the most trustworthy regions in input masks during transitions between stages. For instance, the detection step can give more weight to well-segmented areas. During reconstruction, attention maps can highlight tumors confirmed in earlier steps. This focus on reliable regions helps prevent noisy or inaccurate data from being amplified in later predictions.

The conceptual contributions of the study are profound, advancing the domain of medical computer vision, particularly in the use of machine learning for lung cancer detection. By incorporating advanced algorithms like GANs, off-policy PPO, and TLSTM, our model overcomes traditional image segmentation and tumor detection challenges, establishing a new benchmark for diagnostic performance. This research enhances our knowledge of how various machine learning techniques can work together effectively to address specific challenges in medical imaging, including balancing skewed data distributions and improving the relevance of features. The success of our model may encourage further research into combining these technologies, potentially expanding their use in other medical diagnostic and treatment planning areas. Our findings also suggest a new avenue for developing machine learning frameworks capable of handling the complexities of real-world medical data, opening up possibilities for creating more personalized and precise medical treatment strategies.

The limitations of the proposed model are as follows:

- Dependency on high-quality and well-annotated data: A significant limitation of our model is its dependency on high-quality, well-annotated imaging data. Access to such diagnostic images and annotations can be inconsistent in medical imaging, especially in underdeveloped regions or institutions with limited resources. This inconsistency could lead to discrepancies in the performance of the model, potentially affecting its reliability when used in various clinical environments. The reliance on detailed annotations for training introduces challenges, as any inaccuracies or omissions in annotations can misguide the learning process and result in erroneous interpretations or overlooked detections. Developing semi-supervised learning algorithms that require fewer labeled examples could mitigate this issue. Additionally, collaborating with multiple institutions to diversify training datasets would help the model generalize better across different settings and equipment variations.
- Variation in imaging equipment and acquisition parameters: A key limitation lies in the model’s untested generalizability across different imaging devices and acquisition protocols. Differences in scanner vendors, reconstruction kernels, radiation doses, and slice thicknesses can significantly alter image characteristics. As the current model was primarily trained on data from a single or limited range of imaging protocols, its performance may degrade when applied to datasets acquired under different technical conditions. Future work should include training and validation using multi-center data from diverse acquisition settings to enhance robustness and adaptability.
- Computational resource intensity: The complex architecture of our model, which includes multiple advanced algorithms such as GANs, off-policy PPO, and deep learning networks, is computationally intensive. This demands significant computational resources, which might not always be available in all real-time clinical settings, particularly in resource-limited environments. High computational demands may slow processing times, making it impractical in clinical settings where rapid diagnostics are crucial. To overcome this limitation, optimizing the model to reduce its computational load without significantly sacrificing performance could be beneficial. Techniques such as model pruning, quantization, and employing more efficient network architectures could be considered. Additionally, leveraging cloud computing resources could provide a viable solution by offloading heavy computational tasks to the cloud, thus reducing the need for extensive local computing resources.
- Requirement for extensive training data: Training a deep learning model from scratch requires a vast amount of data to achieve satisfactory performance, posing a significant barrier, especially when addressing rare conditions or settings where data collection is challenging. This requirement can also prolong training time and increase computational resource needs. Transfer learning techniques can address this limitation, where a model developed for one task is repurposed for another. This method enables the model to utilize pre-trained networks that have already captured essential features from extensive and varied datasets, thereby lessening the data needed for training. Additionally, generating synthetic data through techniques like GANs can augment real datasets with synthetic examples, providing more comprehensive training material and enhancing the robustness of the model without requiring vast real datasets.
- Model complexity and interpretability: The high complexity of the model presents challenges in interpretability, which is crucial in medical applications where practitioners need to understand how the model makes diagnostic decisions. Complex models like GANs and deep reinforcement learning algorithms can obscure the decision-making process, potentially causing trust issues among clinicians. Enhancing the interpretability of the model could involve integrating techniques such as feature visualization, allowing users to see what the model focuses on when making decisions. Additionally, simplifying the model without significantly compromising performance and creating interfaces that clearly explain the decision-making process to clinicians can enhance the transparency and trustworthiness of these models.
- Generalization across different demographics: The capacity of the method to generalize across various patient demographics and geographic locations is limited. Lung tissue characteristics, which can vary significantly due to ethnicity, age, and environmental influences, may affect the applicability of the model. To improve the generalization capabilities of the model, training it across a more diverse set of imaging data representing different demographics would be beneficial. Incorporating adaptive algorithms that adjust their parameters based on the demographic data of patients could enhance the effectiveness of the model in diverse clinical settings. This approach would help ensure the model remains robust and reliable regardless of deployment location, ultimately leading to better patient outcomes globally.

## Conclusion

This study presents a significant advancement in lung cancer diagnostics by developing a novel computational framework capable of generating 3D representations of lung tumors from CT scans. By incorporating a custom GAN enhanced with off-policy PPO, our method successfully tackles the challenge of accurately segmenting lung tissue by overcoming the bias caused by a high proportion of non-lung pixels. This method increases the effectiveness of segmentation and enhances the detection of minority samples using a novel reward system. Furthermore, incorporating a specialized GAN with a new loss function significantly advances tumor detection within these accurately segmented areas. After segmentation and detection, the EfficientNet model extracts critical features, refined by a spatial attention-based TLSTM network. This improvement is vital for enhancing the performance of 3D reconstructions by concentrating on adjacent samples within a transductive learning framework, thus achieving exceptional precision. Empirical validation of our model on the LIDC-IDRI dataset has demonstrated superior performance, achieving HD and ED metrics of 0.648 and 0.985, respectively. These findings underscore the potential of our framework to serve as a valuable clinical tool, significantly enhancing the capabilities of radiologists in the diagnosis and treatment planning of lung cancer, ultimately aiming to improve patient outcomes. This study not only contributes to the technical fields of image processing and machine learning but also holds profound implications for clinical practice, emphasizing the importance of advanced computational tools in enhancing the effectiveness of medical diagnostics in oncology.

Future work could focus on integrating this advanced framework with real-time imaging systems to facilitate live diagnostics during clinical procedures. This would allow radiologists and surgeons to view 3D representations of lung tumors in real-time, potentially during surgical planning or interventional procedures. Such integration could improve the quality of interventions and reduce the need for multiple imaging sessions, thereby enhancing patient management efficiency. Adapting the model to function in real-time will involve optimizing the algorithms for faster processing speeds without sacrificing the reliability of tumor detection and segmentation. Another area for future work is expanding the application of this framework to other types of cancer and complex diseases, where imaging plays a crucial role in diagnosis and treatment planning. The utility of the model could be significantly broadened by customizing the model to handle different kinds of tissue characteristics and disease markers found in other cancers, such as breast, prostate, or brain cancers. This would require adapting the current algorithms to recognize and process the unique patterns and features of different disease states, potentially helping to identify and manage a broader range of conditions.

## Data Availability

The datasets used and/or analysed during the current study are available from the corresponding author on reasonable request.
